# A global evaluation of the effectiveness of voluntary REDD+ projects at reducing deforestation and degradation in the moist tropics

**DOI:** 10.1111/cobi.13970

**Published:** 2022-09-08

**Authors:** Alejandro Guizar‐Coutiño, Julia P. G. Jones, Andrew Balmford, Rachel Carmenta, David A. Coomes

**Affiliations:** ^1^ Department of Plant Sciences and Conservation Research Institute University of Cambridge Cambridge UK; ^2^ School of Natural Sciences College of Engineering and Environmental Sciences Bangor University Bangor UK; ^3^ Department of Zoology and Conservation Research Institute University of Cambridge Cambridge UK; ^4^ Tyndall Centre and School of International Development University of East Anglia Norwich UK

**Keywords:** carbon, ecosystem services, forest loss, impact evaluation, matching, nature‐based solutions, Carbono, correspondencia, evaluación de impacto, pérdida de bosques, servicios ecosistémicos, soluciones basadas en la naturaleza, 碳, 森林丧失, 生态系统服务, 基于自然的解决方案, 影响评估, 匹配

## Abstract

Reducing emissions from deforestation and forest degradation (REDD+) projects aim to contribute to climate change mitigation by protecting and enhancing carbon stocks in tropical forests, but there have been no systematic global evaluations of their impact. We used a new data set for tropical humid forests and a standardized evaluation approach (based on pixel matching) to quantify the performance of a representative sample of 40 voluntary REDD+ projects in 9 countries certified under the Verified Carbon Standard (VCS). In the first 5 years of implementation, deforestation within project areas was reduced by 47% (95% confidence interval [CI]: 24–68) compared with matched counterfactual pixels, and degradation rates were 58% lower (95% CI: 49–63). Reductions were small in absolute terms but greater in sites located in high‐deforestation settings and did not appear to be substantially undermined by leakage activities in forested areas within 10 km of project boundaries. At the 26th Conference of the Parties of the United Nations Framework Convention on Climate Change, the international community renewed its commitment to tackling tropical deforestation as a nature‐based solution to climate change. Our results indicate that incentivizing forest conservation through voluntary site‐based projects can slow tropical deforestation and highlight the particular importance of prioritizing financing for areas at greater risk of deforestation.

## Abstract (es)

Evaluación Global de la Efectividad de proyectos REDD+ en la Reducción de la Deforestación y Degradación en el Trópico Húmedo

**Resumen:**

Los proyectos para la reducción de emisiones derivados de la deforestación y degradación de bosques (REDD+) tienen como meta contribuir a la mitigación del cambio climático al protejer y  fomentar la disponibilidad de carbono en bosques tropicales, pero a la fecha no se han realizado evaluaciones globales sistemáticas sobre su impacto. Utilizamos bases de datos recientes sobre bosques tropicales húmedos y un método estandarizado de evaluación (basado en ‘emparejamiento’ [matching] de pixeles) para cuantificar el desempeño de una muestra representativa de 40 proyectos voluntarios REDD+ , localizados en 9 países y certificados bajo el estándar Verified Carbon Standard (VCS). En los primeros 5 años de implementación, la deforestación en las áreas de los proyectos disminuyó en 47% (IC 95% 24–68) en comparación con los pixeles contrafactuales correspondientes, y las tasas de degradación fueron 58% menos (IC 95% 49–63). Las reducciones fueron pequeñas en términos absolutos pero mayores en sitios con tasas de deforestación elevadas, y no parecieron ser afectadas sustancialmente por efectos de fuga (leakage) en áreas boscosas en un radio de 10 km de los límites del proyecto. En la COP26, la comunidad internacional renovó su compromiso de afrontar la deforestación tropical como una solución al cambio climático basada en la naturaleza. Nuestros resultados indican que incentivar la conservación de bosques mediante proyectos locales voluntarios puede disminuir la deforestación tropical, y resaltan la importancia de priorizar financiamiento en áreas con un mayor riesgo de deforestación.

## 【摘要】

随着世界范围内人类与野生动物的冲突不断升级, 对野生动物的宽容和接纳等概念变得越来越重要。然而, 当代保护研究表明, 人们对人类与野生动物积极互作的认识有限, 导致可能难以准确描述人类与动物的相遇。如果不解决这些局限性, 就会导致野生动物和景观管理计划的设计和实施存在不足, 以及对本土生态知识的否定。我们利用印度喀拉拉邦瓦亚纳德野生动物保护区森林中的阿迪瓦西族Kattunayakans部落的民族志证据, 来研究印度原住民对于人类与野生动物共存的观点。通过定性的实地研究(包括访谈和在森林样带调查), 我们发现Kattunayakans部落表现出对野生动物的宽容和接纳, 其特点是深入的共存, 包括三个中心思想:野生动物是有理性的交流者;野生动物是神、老师和平等的族群;野生动物是实行 “*dharmam*”的具有共同起源的亲戚。我们认为, 充分理解以上几点有助于将Kattunayakan部落的观点引入印度的森林管理, 并更广泛地解决人类与野生动物的冲突。

## INTRODUCTION

Rapid decarbonization of economies is essential to avert the worst impacts of human‐induced climate change but protecting natural ecosystems and the carbon they store is also necessary (Griscom et al., 2017; Seddon et al., 2020). Conserving tropical forests could contribute significantly to achieving net zero emission targets that are needed to limit global warming to below 2°C in the coming decades (Goldstein et al., 2020). If delivered at scale, keeping carbon stored in forests by avoiding deforestation and forest degradation could be one of the most effective nature‐based climate solutions (Griscom et al., 2020). Given that tropical forest ecosystems support the majority of terrestrial biodiversity (Barlow et al., 2018; Lewis et al., 2015), slowing the loss of these vital habitats would also have substantial cobenefits for biodiversity (Di Marco et al., 2018; Watson et al., 2018), particularly if REDD+ (reducing emissions from deforestation and forest degradation) helps conserve threatened forests (Murray et al., 2015). The 26th Conference of the Parties of the United Nations Framework Convention on Climate Change (COP26) saw leaders of over 100 countries that contain over 85% of the world's forests make a commitment to bring deforestation and degradation to an end by 2030 through the Glasgow Leaders’ Declaration on Forests and Land Use (UNFCCC, 2021). This declaration is backed by almost US$20 billion of investments from public and private funds. There is justified skepticism from the international conservation community about the likely realization of this commitment because previous international commitments have failed to deliver, including most recently the New York Declaration on Forests, which aimed to halve deforestation by 2021 (NYDF Assessment Partners, 2020).

Reducing emissions from deforestation and degradation (REDD+) is a multilateral framework for achieving climate change mitigation goals by fostering forest conservation, sustainable management of forests, and enhancement of forest carbon stocks (Agrawal et al., 2011). Its goal is to reduce deforestation and forest degradation by creating financial and institutional mechanisms to deliver genuine emission reductions while benefitting local livelihoods and biodiversity (Holloway & Giandomenico, 2009; Agrawal et al., 2011). Around 50 countries have ongoing REDD+ programs at various stages of development, and over 350 REDD+ projects have been initiated to date (Simonet et al., 2020). These are likely to vary in effectiveness because they are exposed to different drivers of deforestation and forest degradation (Simonet et al., 2020), have differing social objectives (Sills et al., 2014; Carmenta et al., 2020), entail different activities to reduce deforestation, and operate under varying degrees of conditionality (Wunder et al., 2018). In practice, REDD+ project implementation has faced many difficulties (Duchelle et al., 2018; Milne et al., 2019). Nevertheless, if the world is going to meet its renewed commitments to protect tropical forests it is important to learn from REDD+ initiatives to date.

Recently researchers have evaluated the impact of REDD+ and similar interventions on tropical deforestation. Randomized control trials in Africa showed that paying households to reduce deforestation is effective (Jayachandran et al., 2017), whereas unconditional payments are not (Wilebore et al., 2019). Similarly, REDD+ interventions along Brazil's Trans‐Amazon Highway have reduced deforestation rates by 50% relative to matched control sites (Simonet et al., 2019), whereas 2 other studies of Amazonian REDD+ projects showed that voluntary REDD+ projects have little impact (Correa et al., 2020; West et al., 2020). Deforestation rates in Guyana remained below expected levels, whereas a Norway‐supported jurisdictional REDD+ program was active (Roopsind et al., 2019). Given this heterogeneity in results, it seems timely to quantify impacts across a large sample of REDD+ projects. Thus, we quantified the impact of site‐based REDD+ projects on deforestation and forest degradation across a global sample of projects.

## METHODS

### Selection of an initial set of REDD+ projects

A REDD+ project earns carbon credits for independently verified emission reductions relative to a business‐as‐usual scenario (e.g., an estimation of emissions in the absence of the project). These reductions may arise by avoiding deforestation, reducing degradation, or increasing forest cover through reforestation activities. We selected REDD+ sites from the Verified Carbon Standards (VCS) database. The VCS is one of the leading accreditation registries for voluntary REDD+ projects (Donofrio et al., 2019) and one of the only registries that had  geospatial data of REDD+ projects available when project boundary data for this study was collected.

Between January and March 2019, we gathered project design documents, validation reports, and geospatial data sets depicting project area boundaries from the VCS database (http://www.vcsprojectdatabase.org). We focused exclusively on projects categorized as “reducing deforestation and degradation” and established in the tropics (Africa, Southeast Asia, Latin America, and Oceania), of which 81 were found. We contacted project managers and the VCS registry to request source boundary files if project boundary maps were not available. For the 71 projects for which we obtained boundary files, we normalized overlapping polygons so that each overlap was contained by a unique geometry and reprojected the database to a Mollweide equal‐area projection (Bingham et al., 2019).

The VCS methodology constrained how we could analyze the effects of the projects. Specifically, the avoided deforestation protocols require that a project's spatial extent (i.e., its “accounting zone”) contain a parcel of land that has maintained 100% forest cover for at least 10 years prior to the project starting date. Thus, any deforested areas adjacent to or within REDD+ boundaries were systematically excluded from the project area boundaries provided to VCS for monitoring, reporting, and verification purposes (Shoch et al., 2011). Thus, we had to adopt a similar approach and defined our basic unit of analysis as a pixel that was observed to have remained as undisturbed forest from 1990 until the project starting year. This meant we could not employ a difference‐in‐difference approach to isolate the effect of a project because deforestation in the project area was, by definition, zero prior to project commencement. Nevertheless, after‐only analysis is widely used to evaluate the impacts of conservation interventions on environmental outcomes, including deforestation (Rasolofoson et al., 2015; Eklund et al., 2016; Geldmann et al., 2019).

We diverged from VCS protocols in our approach to estimate project additionality. Under VCS, a project must select a counterfactual area of forest that has similar deforestation threats to the project area. We instead adopted a pixel‐based matching approach, which meant pixels were scattered over many sites, instead of a single area. A benefit of this approach is that it ensures that the control set of pixels is exposed to the same geographic drivers of deforestation as the pixels in the REDD+ project sites (Schleicher et al., 2019).

### Yearly maps of forest cover, deforestation, and forest degradation

Annual maps of forest cover, deforestation, and forest degradation were taken from the recently published Tropical Moist Forests (TMF) database (Vancutsem et al., 2021), which was derived from time series of multispectral imagery collected by Landsat. Pixels were of approximately 30‐m resolution. This database provides a long‐term (1990–2019), annual characterization of forest disturbances. We focused our analyses on quantifying temporal changes in 3 forest classes as defined by Vancutsem et al. (2021): *undisturbed*, which represents closed evergreen or semievergreen forest areas that have not been disturbed over the entire period examined; *degraded*, which represents existing or regrowing evergreen or semievergreen forest that has been temporarily disturbed (visible for up to 2.5 years) due to anthropogenic causes, such as selective logging, or from natural causes such as wind storms or fires; and *deforested*, representing long‐term forest disturbances (>2.5 years) and complete removal of forest cover. Forest degradation is commonly defined as a loss of productivity and a reduction of forest biomass due to anthropogenic and natural causes (Thompson et al., 2013). However, forest degradation in the TMF database refers to events that substantially but temporarily alter pixel spectral characteristics. These events may include subpixel changes, such as the opening of small logging roads, the majority (64%) of which are of <6 months duration (Vancutsem et al., 2021). Such disturbances may be associated with significant loss of forest carbon stocks and yet appear transient in optical imagery because canopies rapidly regain greenness. Hence, what appear as short‐term disturbance events in the optical imagery may have enduring impacts in carbon stocks and forest structure (Rappaport et al., 2018). However, the relationship between degradation events as characterized in Vancutsem et al. (2021) and biomass loss has yet to be quantified.

The 71 REDD+ sites for which we had boundary maps were established in moist and seasonally dry tropical biomes, but because the TMF database does not contain data on drier regions, we limited our analyses to sites that were densely forested (i.e., at least 80% forest cover at the project start date), which brought the total down to 54 projects. Furthermore, because our estimation approach involved analyzing change in forest cover for at least 5 years after project implementation (see “Impacts of REDD+ projects on deforestation and forest degradation rates”), we excluded 3 projects that had been active for shorter periods. We also excluded 1 site that started operations before the year 2000 (because this is prior to the operationalization of REDD+), leaving us with 50 projects with which to search for counterfactual observations for comparison (see “Matching”).

### Sampling design in project areas and surrounding landscapes

We used a pixel sampling approach to characterize project areas (i.e., treatment areas) and the regions where these were located, from which we identified control groups to evaluate treatment effects. Pixels in the treatment areas were sampled by creating a regular pattern of sampling points, each separated by 250 m, within the boundaries of REDD+ projects based on the project boundary files. To generate observations from which we generated control pixels by matching (see below), a large number of pixels (up to 7 times the number in the project area) located in the same country and biome as treatment pixels were sampled at random. We retained pixels if they remained undisturbed for at least 10 years prior to the project starting date (i.e., mirroring the VCS method for project areas). Following this approach, all the control and treatment pixels we used had zero deforestation and degradation rates in the 10 years up to project implementation.

To account for local leakage effects in our design, we defined 10‐km areas around the REDD+ interventions from which we did not sample pixels for matching (hereafter leakage belts). Leakage occurs when deforestation activities in project areas are shifted elsewhere upon project implementation and is a widely acknowledged risk of REDD+ and other forest‐based interventions (Pfaff & Robalino, 2017). We also evaluated evidence of local leakage by estimating changes in forest cover within 10 km immediately outside project boundaries after project implementation (Ewers & Rodrigues, 2008). Leakage belts were adjusted to exclude overlaps with protected areas, other nearby REDD+ projects, and overlaps between buffer zones of neighbouring REDD+ projects (*n* = 7) (Appendix S6). We then assessed the extent to which leakage activities took place (e.g., significant differences in deforestation rates) by examining deforestation patterns before and after project implementation within leakage belts (see “Quantifying local leakage”).

### Matching

We performed statistical matching to identify sets of control pixels for each project area that were similar in observable confounders associated with forest loss, thus ensuring that selected controls were exposed to the same drivers of deforestation as project area pixels. To implement a standardized method, we sought a single set of covariates across the full set of sites, acknowledging that drivers of deforestation vary across the countries included in this study (Curtis et al., 2018). We collected pixel‐level data on sociodemographic and biophysical characteristics that are typically associated with deforestation (Angelsen & Kaimowitz, 2001; Busch & Ferretti‐Gallon, 2017): elevation and slope (Jarvis et al., 2008), distance to the nearest urban center in 2015 (Weiss et al., 2018), and distance to forest edge (Laurance et al., 2011). To account for temporal changes in distance to forest edge, we constructed annual time series of the mean distance to the nearest deforested pixels based on the TMF map. For each sampled pixel, we calculated the distance to the closest pixel that had changed its status from undisturbed to deforested or from degraded to deforested during the observed year (for 2000–2019). We then produced a rolling average estimate of the mean distance to the closest deforested pixel in the previous 5 years for 2005–2019.

**FIGURE 1:**
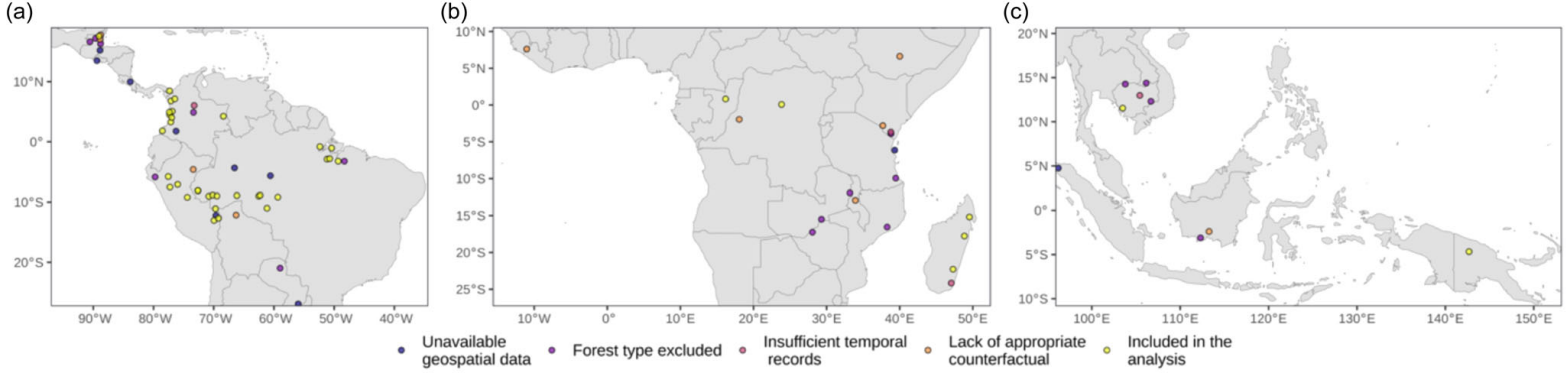
Location of the 81 verified carbon standards projects in (a) the Americas, (b), Africa, and (c) Asia and Oceania analyzed for project effectiveness at reducing deforestation and degradation (blue dots, 10 projects without detailed maps of locations; purple, 17 projects with <80% evergreen forest cover at the start; pink, 4 projects operating for fewer than 5 years or commencing before 2000; orange, 10 projects that could not be matched with appropriate control pixels; yellow, 40 projects in the impact evaluation)

Matching was performed with the R MatchIt package (Ho et al., 2011). We measured the similarity between treatment and control pixels with the Mahalanobis distance metric (Legendre, & Legendre, 1988), which is effective in producing balanced comparison groups when the number of matching covariates is relatively low (Stuart, 2010). We performed 1:1 nearest‐neighbour matching with replacement using elevation, slope, mean distance to population centres and mean distance to deforested areas over the five years prior to project commencement (Appendix S7). We used exact matching on country and terrestrial biome, as defined by Dinerstein et al. (2017). For 10 sites that intersected more than one terrestrial biome (e.g., broadleaf forests and mangroves), we subdivided REDD+ sites to generate sets from the same biome to match against controls. By matching within the same tropical moist forests in the same biomes and countries, we ensured comparability of bioclimatic conditions for agricultural development. We considered an absolute standardized mean difference of <0.25 between treated and control samples across all covariates as acceptable (Stuart, 2010). Only those REDD+ projects that met this criterion for at least 90% of pixels (across all subgroups) were included in further analyses. Ten sites were dropped after matching because they were not matched across all covariates (Figure 1; Appendix S14). To evaluate whether the resulting subset of 40 sites was representative of the socio‐environmental conditions in the original set of 71 sites, we ran a logistic regression to predict the probability of being included in this analysis as a function of accessibility, distance to deforestation by project staring date, elevation, human development index, and project area size. If socio‐environmental conditions in the filtered data set were different from those in the original data set, we expected significant effects in the model (having applied a Bonferroni correction for multiple comparisons). Furthermore, we ran a post hoc analysis of the importance of the covariates we used for matching that confirmed that they did predict forest loss. The final parsimonious model described a moderate proportion of the observed variation in deforestation across the examined landscapes (Nagelkerke's *r* ^2^ = 0.47) (Appendix S1).

Selecting an appropriate control to evaluate the impact of conservation interventions can be complicated by the presence of other interventions occurring in the landscapes (Schleicher et al., 2019). To account for the presence of protected areas, we ran a separate set of analyses in which we excluded pixels in protected area polygons (Appendix S5), based on the World Database on Protected Areas (UNEP‐WCMC & IUCN, 2019). We standardized the protected area database by removing areas categorized as “not designated” or “inscribed” and UNESCO Biosphere Reserves (Bingham et al., 2019) and reprojected the geometries to a Mollweide equal‐area projection.

### Impacts of REDD+ projects on deforestation and forest degradation rates

Annual deforestation and forest degradation rates were calculated for each REDD+ site and its control pixels. To do this, we built a time series of annual rates of deforestation events spanning 2001 to 2019 for all our groups of treatment and matched control pixels. We then estimated annual deforestation rates within a REDD+ project as $r_{t}=\Delta p_{t}/p_{d}$, where $\Delta p_{t}$ is the total number of pixels deforested in year *t* and *p*_d_ is the number of forested pixels at the start of that project. Annual deforestation rates within control pixels were calculated the same way. We then calculated degradation rates using the same approach in control and treatment sites. Although the TMF database does not differentiate between natural and anthropogenic disturbances, one can expect natural and anthropogenic disturbances to be similarly prevalent in treated and control sets because of our matching approach. These time series provided the information needed to calculate annual deforestation and forest degradation rates from the project starting date for up to 10 years after implementation (where enough data were available).

Absolute differences in deforestation and forest degradation rates between treatments and controls were calculated as ${\bar{r}}_{t}-{\bar{r}}_{c}\;$for each REDD+ site, where $\bar{r}$ is the mean deforestation rate within the first 5 years of implementation and *t* and *c* subscripts refer to treatment and control groups, respectively. We used the 40 site‐level estimates to derive the global mean change in deforestation and estimated 95% confidence intervals by nonparametric bootstrapping. The same approach was used to calculate site‐level differences and global mean change in forest degradation rates. Although we did not incorporate spatial autocorrelation in our analyses of effect sizes, we did reduce autocorrelation in our data sets by taking a systematic sample of pixels within treated areas (each 30‐m pixel separated by 250 m) and by drawing random candidate control pixels from other areas of tropical moist forest in the same country and terrestrial biome (excluding leakage belts) prior to matching. Subsequent analyses to evaluate changes in effect size over time were performed with subsets of sites with enough observations to estimate the treatment effect after 8 and 10 years of implementation. We used all the temporal observations available at each time subset to estimate the treatment effect.

Site‐level proportional differences in forest disturbances were calculated by dividing the mean disturbance rate (e.g., deforestation or forest degradation) at treated sites within the first 5 years of implementation by the mean disturbance rate at control groups over the same period $\left(\frac{{\bar{r}}_{t}}{{\bar{r}}_{c}}\right)$. The overall mean deforestation and forest degradation across the 40 sites was calculated to estimate the proportional reduction in disturbance rates associated with REDD+ projects globally; 95% confidence intervals (CIs) were estimated by bootstrapping.

### Effectiveness of REDD+ in relation to background deforestation rates

Given that the sampled REDD+ projects were located in countries and periods with different rates of deforestation, we explored how the REDD+ treatment effect varied with background deforestation. Background deforestation was defined as the mean country‐level tropical moist forest loss rate for the first 5 years of project operation, for the country in which a project was located (Appendix S8). These rates were used to classify projects into high‐ and low‐threat categories, depending on whether the rates were above or below the mean annual deforestation rate observed across the humid tropics over the last 3 decades (i.e., 0.57%/year) (Vancutsem et al., 2021). To determine changes in annual forest loss rates between high‐ and low‐deforestation groups, we grouped site‐level mean differences and derived group‐level mean estimates with 95% CIs with bootstrapping. We conducted a Wilcoxon's rank‐sum test to compare differences in reductions between high‐ and low‐threat groups. To determine threat‐level proportional differences between treatment and controls, we grouped site‐level proportional changes and high‐ and low‐deforestation groups and derived mean estimates with 95% CIs by bootstrapping. We repeated the analyses to derive absolute and proportional changes in forest degradation between high‐ and low‐deforestation groups.

### Quantifying local leakage

We evaluated local leakage by testing whether there was a change in annual deforestation rates in the 10‐km leakage belts (e.g., buffer zones) following project implementation. Annual deforestation rates within leakage belts were estimated by dividing the area deforested for each year (1991–2019) by the extent of undisturbed forests in 1990. We extracted 5 years of data before and after projects started and tested whether there was an increase in rate following implementation with site‐level bootstrapped *t* tests. We examined whether leakage increased or decreased over time by performing bootstrapped *t* tests on subsets of sites with enough observations to examine changes over 8 and 10 years before and after project implementation.

## RESULTS

### Project selection

The 40 REDD+ projects selected for this study, following a systematic filtering of the initial database, were located in 9 countries and together encompassed 8.38 million ha of humid tropical forest, with a median area of 92,353 ha (interquartile range = 46,192–190,660 ha). Thirty‐three were in the Americas, 5 in Africa, 1 in Asia, and 1 in Oceania (Figure 1). Several projects in Africa and Asia were excluded because they were situated in dry forest and savanna regions into which the TMF deforestation maps do not extend. Although the analyzed sites represented a subset of the 71 projects initially obtained from the VCS database examination, they were similar to the wider sample in most characteristics but were significantly closer to populated centers (Appendix S17). This suggests our analyses may be indicative of the performance of VCS REDD+ projects in sites that are more exposed to deforestation.

### Average effectiveness of REDD+ projects

**FIGURE 2:**
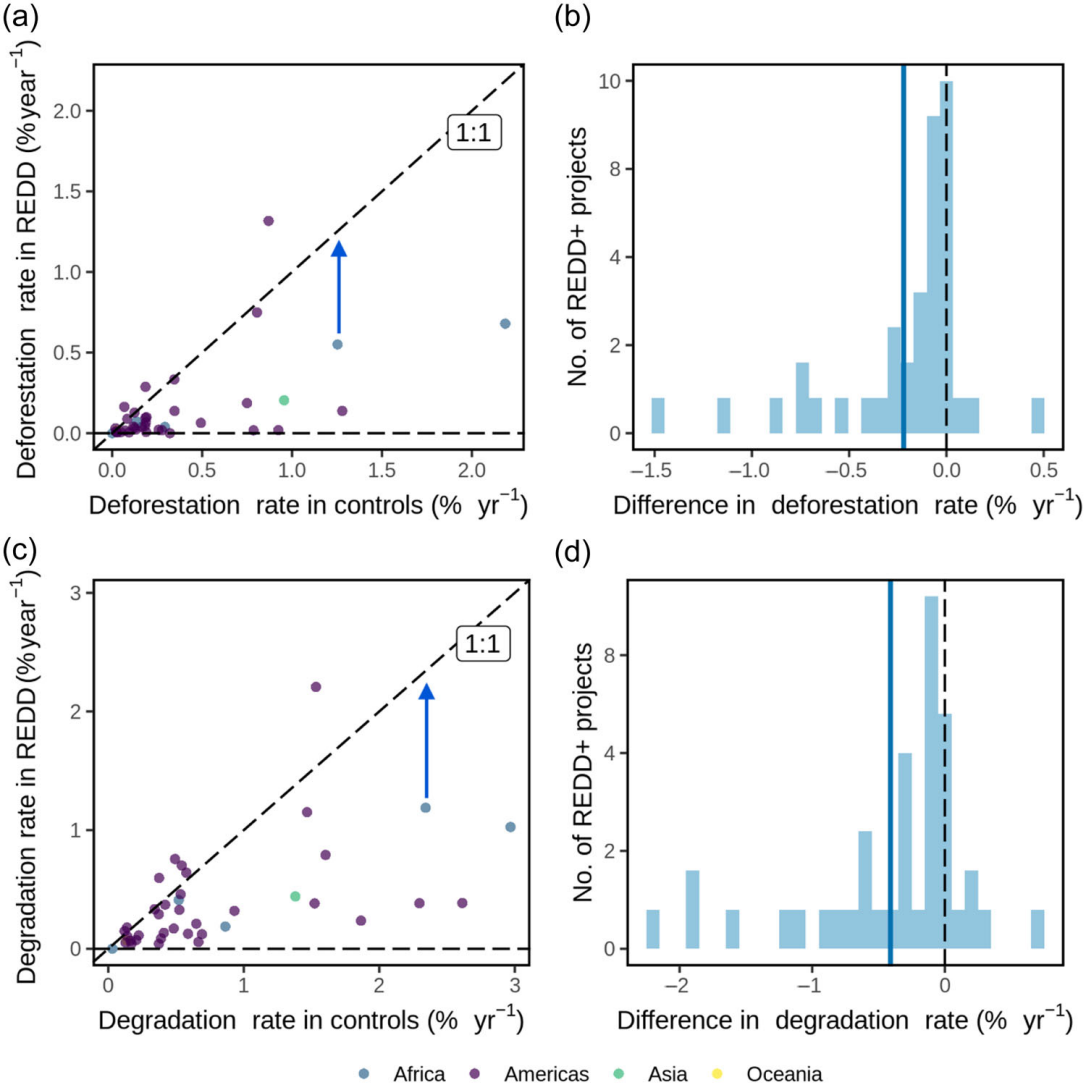
Changes in deforestation and degradation rates resulting from REDD+ projects over their first 5 years of operation: (a, c) deforestation and degradation rates in REDD+ projects versus matched control pixels (blue arrows, change in deforestation or degradation resulting from a project is the vertical distance between the data point and the diagonal 1:1 line) and (b, d) differences in deforestation and degradation rates relative to controls (vertical blue and dashed lines over the zero threshold; mean)

We found REDD+ project implementation was associated with reductions in deforestation and forest degradation over the first 5 years of operation compared with matched control pixels in the wider landscape (Figure 2). Deforestation rates decreased in 34 sites, and small increases occurred in 6 sites (Figure 2a). Mean reduction was 0.22%/year (95% CI: 0.13–0.36) (Figure 2b) compared with matched control mean deforestation rates of 0.36%/year (0.25–0.55). Reductions in degradation rates occurred in 33 sites. Small increases in degradation occurred in 7 sites, and increased deforestation occurred in 4 of these (Figure 2c). Estimated mean reduction was 0.41%/year (0.24–0.65) relative to matched controls mean degradation rates of 0.80%/year (0.60–1.00).

**FIGURE 3:**
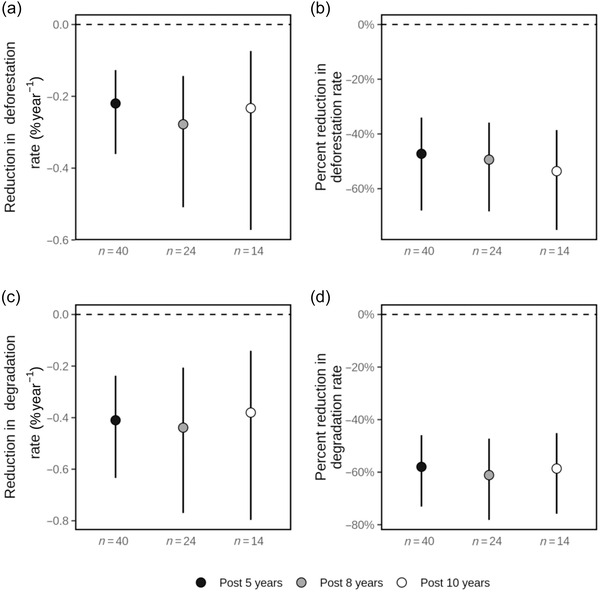
In 40 REDD+ projects for 3 postimplementation periods (a, c) reductions in annual deforestation and degradation rates and (b, d) percent reductions in deforestation and degradation rates (means and 95% confidence intervals [CIs])

Expressing changes in deforestation or degradation as relative reductions (i.e., as a percentage of rates observed in controls), REDD+ projects reduced deforestation by 47% (95% CI: 24–68) and degradation by 58% (49–63) in the first 5 years (Figure 3). These annual reductions in deforestation rates amounted to a total of 66,754 ha of avoided forest loss, and the annual reduction in degradation amounted to 116,910 ha of avoided forest degradation across all 40 project sites within the first 5 years of project implementation, which equated to ∼0.8% and ∼1.4%, respectively, of the combined area of these REDD+ projects. Rates of deforestation and degradation were closely correlated among projects (Spearman's *ρ* = 0.82, *p* < 0.0001) (Appendix S9), and degradation occurred at roughly twice the rate of deforestation.

When examining the subset of projects that had been operating for at least 8 and 10 years (*n* = 24 and 14, respectively), we found no evidence of varying effect sizes through time because we observed similar estimates of reductions in deforestation (Figure 3a) and degradation (Figure 3c) throughout these periods. Moreover, estimates of avoided deforestation and forest degradation were hardly affected by whether we included or excluded protected areas from our matching analyses. (Appendices S2–S4). Mean reduction of deforestation rates was 0.30%/year (95% CI: 0.18–0.47), and mean reduction in degradation rates was 0.49%/year (0.30–0.74) relative to matched controls pixels outside protected areas.

### Variation in REDD+ effectiveness in relation to background deforestation rates

**FIGURE 4:**
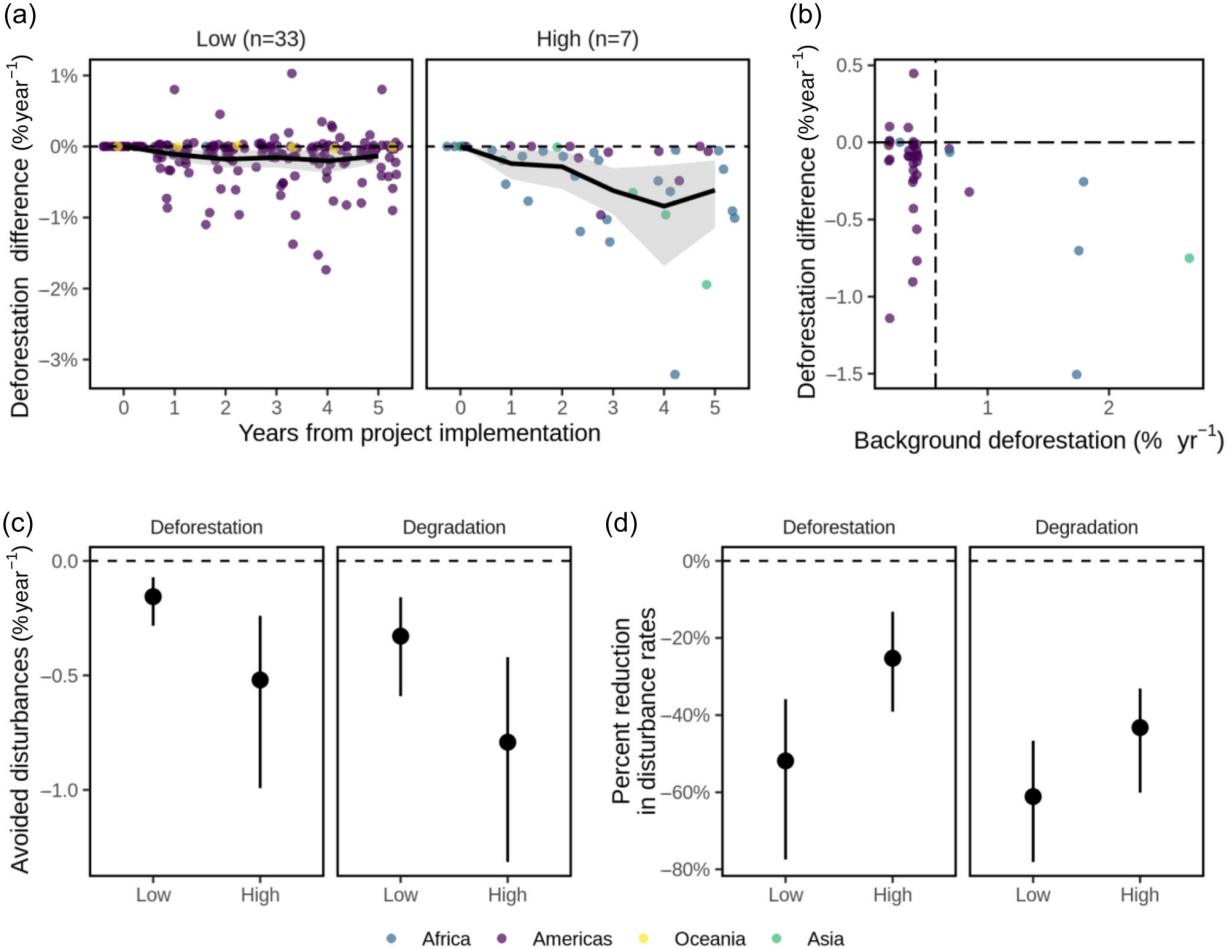
Effectiveness of REDD+ projects relative to background deforestation rates for 40 REDD+ project sites in humid tropical forests: (a) annual differences in deforestation (with jitter) between project areas and matched controls over 5 years after project implementation (black line, mean annual differences; shading, 95% confidence interval [CI]); (b) mean differences in deforestation rates relative to country‐level background deforestation rates in the humid tropics (calculated for the project implementation period) (vertical line, pan‐tropical mean rate of deforestation [0.57%/year]); (c) mean differences in deforestation and degradation rates in regions categorized as having low (<0.57%/year) or high (>0.57%/year) deforestation rates based on average deforestation rate across the entire humid tropics; and (d) mean percent reductions in deforestation and degradation rates relative to controls in regions of high and low deforestation rates. The 95% confidence intervals (CIs) in panels (a), (c), and (d) are based on nonparametric bootstrapping.

Country‐level background deforestation rates and reductions in deforestation (Spearman's *ρ* = 0.42, *p* = 0.006) (Figure 4b) and reductions in forest degradation were moderately correlated (*ρ* = 0.39, *p* = 0.013) (Appendix S10). The REDD+ projects in the low‐threat group showed small reductions in deforestation (mean = 0.16%/year [95% CI: 0.07–0.28]) and degradation (mean = 0.33%/year [0.16–0.58]) rates. Substantially greater effect sizes were observed for the 7 projects in the high‐threat group: deforestation was reduced by 0.52%/year (0.25–1.0) and degradation by 0.79%/year (0.42–1.32) (Figure 4c). We calculated that 49,197 ha of forests saved by REDD+ projects were in regions of high threat (i.e., 74% of the total saved), even though these forests only represented 20.5% of the total area of the 40 projects investigated. Therefore, in the high‐threat group, ∼2.9% of the area of REDD+ projects was saved over the first 5 years. When measuring relative reductions in forest disturbances, we observed larger effect sizes in low‐threat groups compared with high‐threat groups. Mean reductions in deforestation were 52% (36–76) and 25% (13–39), and mean reductions in degradation were 61% (47–79) and 43% (33–60) in the low‐ and high‐threat groups, respectively (Figure 4d).

### Evidence of local leakage

**FIGURE 5:**
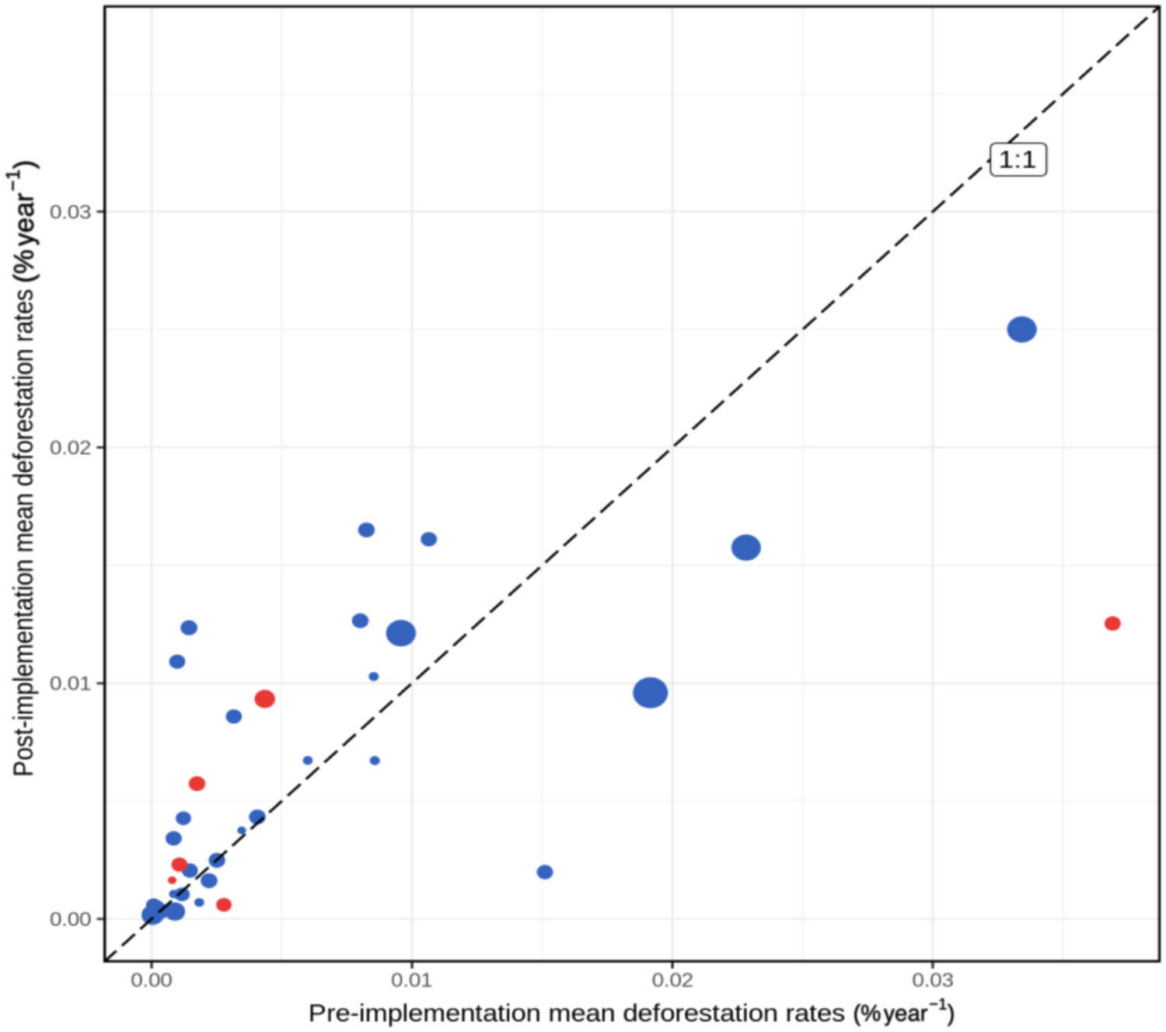
Mean deforestation rates 5 years after versus 5 years before REDD+ project commencement in the 10‐km leakage belt (i.e., deforestation displacements due to project implementation) (red circles, differences in postimplementation deforestation rates relative to the preimplementation period [bootstrapped *t* tests, *p* < 0.05]; blue circles, differences not statistically significant; circle sizes, scaled to reflect background deforestation rates observed at the host country within the first 5 years of project implementation)

There was no evidence of local leakage of deforestation activities from project areas within the 10‐km leakage belts following project implementation. Within 5 years of project implementation, 3 sites had higher rates of deforestation in the leakage belts after project implementation, whereas 2 sites had lower rates (bootstrapped *t* tests, *p* < 0.05) (Figure 5). When examining the variation in leakage effects on subset of projects operating for at least 8 years, we found 1 project with higher rates of deforestation, whereas 4 sites showed a reduction in deforestation (Appendix S11a). Deforestation rates relative to leakage belts did not change in the subset of projects operating for at least 10 years (Appendix S11b).

## DISCUSSION

Across 40 voluntary REDD+ projects in 9 countries, on average REDD+ interventions reduced deforestation and degradation relative to control pixels over the first 5 years of operation. The projects achieved greater reductions in deforestation and forest degradation where the threat of deforestation was greatest. We also found consistent reductions in deforestation and forest degradation when comparing the effectiveness of REDD+ relative to matched pixels outside protected areas. The absolute reductions in these rates were modest in most projects, but in relative terms both rates were roughly halved by the projects we investigated. The REDD+ projects did not always deliver reductions in deforestation and degradation. Deforestation increased at 6 sites and forest degradation increased at 7 sites, and 4 sites showed an increase in both deforestation and degradation. Although effect sizes were close to zero for the majority of these sites, our results illustrate the challenges of REDD+ implementation.

To the best of our knowledge, ours is the first study in which remotely sensed degradation and deforestation data were used to test whether voluntary REDD+ projects are effective at reducing small‐scale temporary disturbances, alongside long‐term deforestation, across a sample of geographically dispersed projects that differ in deforestation drivers and social objectives. Protecting and restoring natural forests could be a nature‐based climate solution that is cost‐effective and could have substantial impact if the many hurdles to implementation can be overcome (Duchelle et al., 2018; Milne et al., 2019; TSVCM, 2021). Moreover, avoiding deforestation and forest degradation is paramount for safeguarding biodiversity and can play a role in safeguarding noncarbon ecosystem services, such as water regulation and soil productivity (Griscom et al., 2017). Our results provide some room for optimism. Despite the many challenges to just and economically sustainable implementation, the initial wave of REDD+ projects were effective at reducing forest loss. The 66,754 ha of spared forests over the first 5 years is small; indeed, it is smaller than the median size of the examined REDD+ sites (median = 92,353 ha) and much smaller than a typical protected area. Nevertheless, with pressures on biodiverse tropical forests expected to grow in the future (Laurance et al., 2014; Barlow et al., 2018), the evidence that REDD+ has reduced deforestation and degradation where the threat of deforestation was greatest is particularly important. Moreover, the finding that reductions in deforestation and forest degradation were still evident after excluding protected areas from the matching analyses suggests a positive effect of REDD+ that is not confounded with protected area designation.

Estimating the impact of an intervention, such as REDD+, from observational data is inherently difficult because it relies on estimating what would have happened in the absence of the intervention (Ferraro, 2009; Ferraro & Hanauer, 2014; Baylis et al., 2016). We have matched our REDD+ and control pixels with appropriate drivers of deforestation, but there will inevitably be unobserved covariates. For example, VCS REDD+ projects have often been implemented in areas where conservation nongovernmental organizations have been operating for some time (Sunderlin & Sills, 2012; Usmani et al., 2018) and are likely associated with certain land tenure conditions (Wunder, 2013). This has implications for the selection of appropriate controls and therefore our results. For example, where REDD+ projects are the most recent manifestation of long‐running conservation efforts at sites (Lin et al., 2012; Sunderlin & Sills, 2012), it is not possible to say how much reductions in deforestation is due to the REDD+ specifically, given the long‐term engagement at these landscapes.

Different methods can be used to derive impacts of forest interventions when temporal observations are available for treatment and control groups and before and after project implementation, such as combining matching with difference in differences (Costedoat et al., 2015; Santika et al., 2021) or using synthetic control methods to ensure similar pretreatment deforestation rates between treatment and control groups (West et al., 2020). We used a simpler approach: matching units with similar modeled deforestation risk without pretreatment comparisons (e.g., Rasolofoson et al., 2015; Eklund et al., 2016; Geldmann et al., 2019). Although this approach has limitations (Schleicher et al., 2019), it was most appropriate for our context because VCS requires projects, when delimiting their boundaries, to exclude from the accounting area locations where deforestation has taken place in the 10 years prior to the project start date (Shoch et al., 2011). Therefore, the rates of deforestation in the treatment groups are by definition zero prior to the commencement of projects due to active exclusion of deforested pixels, and thus we applied the same constrains when selecting candidate control observations. For the same reason, although we could have combined matching with difference in differences, we were restricted to an after‐only analysis because deforestation rates in the before period were zero for both treatment and control groups. Nevertheless, we accounted for pretreatment deforestation rates and related deforestation risks by selecting pixels with similar distance to recent forest clearings, which is a strong predictor of deforestation outcomes in the landscapes we examined.

Most Brazilian REDD+ projects had a small, positive impact on deforestation (avoided deforestation observed in 11 out of 12 sites [Appendices S12 & S18]), whereas a previous work focused specifically on the same sites reported no impact or negative impacts for the majority (figure S3 in West et al. [2020]). These differences are likely to have arisen from differences in the approaches used to construct and match counterfactual areas and used to calculate effect sizes. We used pixels as our unit of analysis, allowing us to restrict deforestation and degradation estimation to forests that were standing at the time of project implementation (in line with VCS requirements). In contrast, West et al. (2020) used georeferenced property boundaries (held in Brazil's Rural Environmental Registry) to construct synthetic controls. This approach uses a weighted combination of untreated property boundaries so that the mean deforestation rates in treated and counterfactual groups were similar in the lead up to project implementation. Further research into the relative merits of different approaches to evaluating the impacts of conservation interventions on deforestation is required.

Leakage is challenging to quantify because it requires characterization of enabling factors, such as labor and market conditions (Pfaff & Robalino, 2017), with which to produce a forecast of potential displacements of deforestation activities. While acknowledging the limitations of our approach, by examining statistical differences in deforestation after projects became implemented, we did not observe strong evidence of systematic leakage effects into the buffer zones adjacent to REDD+ projects. However, leakage can occur across countries, through international market adjustments in response to local restrictions (Meyfroidt & Lambin, 2009), but it is very hard to quantify and was not accounted for in our study.

The importance of slowing tropical deforestation and degradation received much attention at the most recent meeting of the COP26, but the term REDD+ was hardly used. Perhaps the reduction in the use of the term REDD+ is an example of what Redford et al. (2013) call a “conservation fad”; that is, what was seen as an innovative new idea becomes tainted by disappointment with the challenges of real‐world implementation and donors, policy makers, and practitioners essentially reinvent the concept with new terminology. However, it is essential to learn from the last decade of REDD+ implementation. The REDD+ projects have already provided invaluable lessons on the central role of land tenure in implementation (Larson et al., 2013), the necessity of ensuring that the rural poor do not bear the cost of forest conservation efforts (Duchelle et al., 2018; Poudyal et al., 2018; Skutsch & Turnhout, 2020), and the need for effective benefit‐sharing systems and appropriate participation in decision‐making and governance (Luttrell et al., 2013; Milne et al., 2019). Our results also highlight the need to standardize methodologies for establishing baselines with which to evaluate the effectiveness of forest‐based interventions to reduce emissions, a point also made by West et al. (2020). It is currently not possible to establish the aggregate impact of VCS REDD+ projects because the various methodologies used to forecast emissions reductions are incomparable and produce different baseline scenarios (Wilebore, 2015).

As understanding of the carbon stores in tropical forest ecosystems improves (Dargie et al., 2017) and understanding of the feedbacks between tropical deforestation and climate change (Baccini et al., 2017) is gained, the case for tropical forests being central to climate change mitigation efforts grows stronger. Our analysis provides promising evidence that site‐based REDD+ projects have helped reduce deforestation, particularly in areas of high deforestation threat. Yet, emissions reductions in the 40 REDD+ projects analyzed represent a tiny fraction of global emissions. In total, they amounted to about 0.01% of 2018 emissions, or 0.13% of emissions from tropical deforestation in 2013. The need to scale up activities is well recognized. Jurisdictional REDD+ programs operating at regional or national scales following UNFCCC REDD+ framework of 2013 may address some of the major challenges of site‐based REDD+ projects (Duchelle et al., 2019). Most importantly, larger scale efforts may be better placed to address the fundamental challenge of key drivers of deforestation embedded in global and domestic supply chains for commodities, such as beef, palm oil, and soya (Curtis et al., 2018; zu Ermgassen et al., 2020), and in expansion of extractive activities, such as mining (Davis et, al. 2020), and so cannot be effectively tackled with site‐based interventions alone (Delabre et al., 2020). Encouragingly, there is evidence that jurisdictional programs can deliver results. Guyana's national‐level program reduced deforestation loss by 35% from 2010 to 2015 (Roopsind et al., 2019). Applying the lessons from the last few decades to deliver effective and, crucially, equitable reductions in tropical forest degradation and deforestation will be critical if the Glasgow COP26 climate change objectives are to be met.

## Supporting information

Additional information is available online in the Supporting Information section at the end of the online article.
