# Supplementary material for: A global evaluation of the effectiveness of voluntary REDD+ projects at reducing deforestation and degradation in the moist tropics
Source: Conserv Biol. 2022 Sep 8;36(6):e13970. doi: 10.1111/cobi.13970 (PMC10086997; doi:10.1111/cobi.13970)
Supplement: Supplementary file 1 — Additional information is available online in the Supporting Information section at the end of the online article. [file COBI-36-0-s001.docx]

A global evaluation of the effectiveness of voluntary REDD+ projects at reducing deforestation and degradation in the moist tropics

Supplementary Information

### Appendix S1. Predicting deforestation with the matching variables.

A statistical modelling approach was used to check whether our matching variables were important drivers of tropical deforestation. Our model of deforestation drivers based on data pooled from control and treatment pixels across the 40 sites. We randomly sampled 20,000 pixels from our matched dataset and fitted generalised linear models with a Bernoulli distribution to assess the probability of forest loss in relation to elevation, slope, accessibility, distance to forest edge and the score of the six World Bank governance indicators averaged over the period 2010-2018 to account for the different countries involved in the study. Before fitting the models, we investigated potential collinearity among selected covariates and identified high collinearity for HDI, GDP and the WB indicators. We fitted univariate models for collinear variables to predict deforestation and treatment assignment, and selected the one with the best model fit, assessed with the AIC. This led to the inclusion of the world bank indicators in the final model.

Table 1: Collinear variables examined in univariate models for predicting deforestation.

| Name | AIC | BIC |
| --- | --- | --- |
| WB indicators | 1406.54 | 1430.26 |
| GDP | 1449.25 | 1472.96 |
| HDI | 1449.51 | 1473.23 |

Before modelling, we scaled all covariates excepting the WB indicators as these are provided as standardised indices. We removed extreme residuals (n=33) after an initial exploration and determined minor underdispersion (0.89) of the minimal model. A moderate proportion of the observed variation in deforestation across the examined landscapes was explained by thus model (Nagelkerke’s r^2^ 0.47). Of the examined variables, distance to recent forest clearings and distance to population centres had the strongest power when predicting deforestation, with coefficient values of -1.23e+03 and -6.40e-01, respectively (p < 0.005).

Call:
glm(formula = is_defor ~ elevation + slope + access + dist_defor_mean_5 +
wb, family = binomial, data = data)

Deviance Residuals:
Min 1Q Median 3Q Max
-1.2587 -0.2130 -0.0021 0.0000 3.6939

Coefficients:
Estimate Std. Error z value Pr(>|z|)
(Intercept) -1.451e+02 5.669e+00 -25.596 < 2e-16 ***
elevation -2.111e-01 4.360e-02 -4.842 1.29e-06 ***
slope -1.284e-01 4.396e-02 -2.922 0.003481 **
access -6.405e-01 7.050e-02 -9.084 < 2e-16 ***
dist_defor_mean_5 -1.230e+03 4.857e+01 -25.324 < 2e-16 ***
wb 5.679e-02 1.576e-02 3.603 0.000315 ***
---
Signif. codes: 0 ‘***’ 0.001 ‘**’ 0.01 ‘*’ 0.05 ‘.’ 0.1 ‘ ’ 1

(Dispersion parameter for binomial family taken to be 1)

Null deviance: 9899.2 on 20026 degrees of freedom
Residual deviance: 5877.1 on 20021 degrees of freedom
AIC: 5889.1

Number of Fisher Scoring iterations: 15


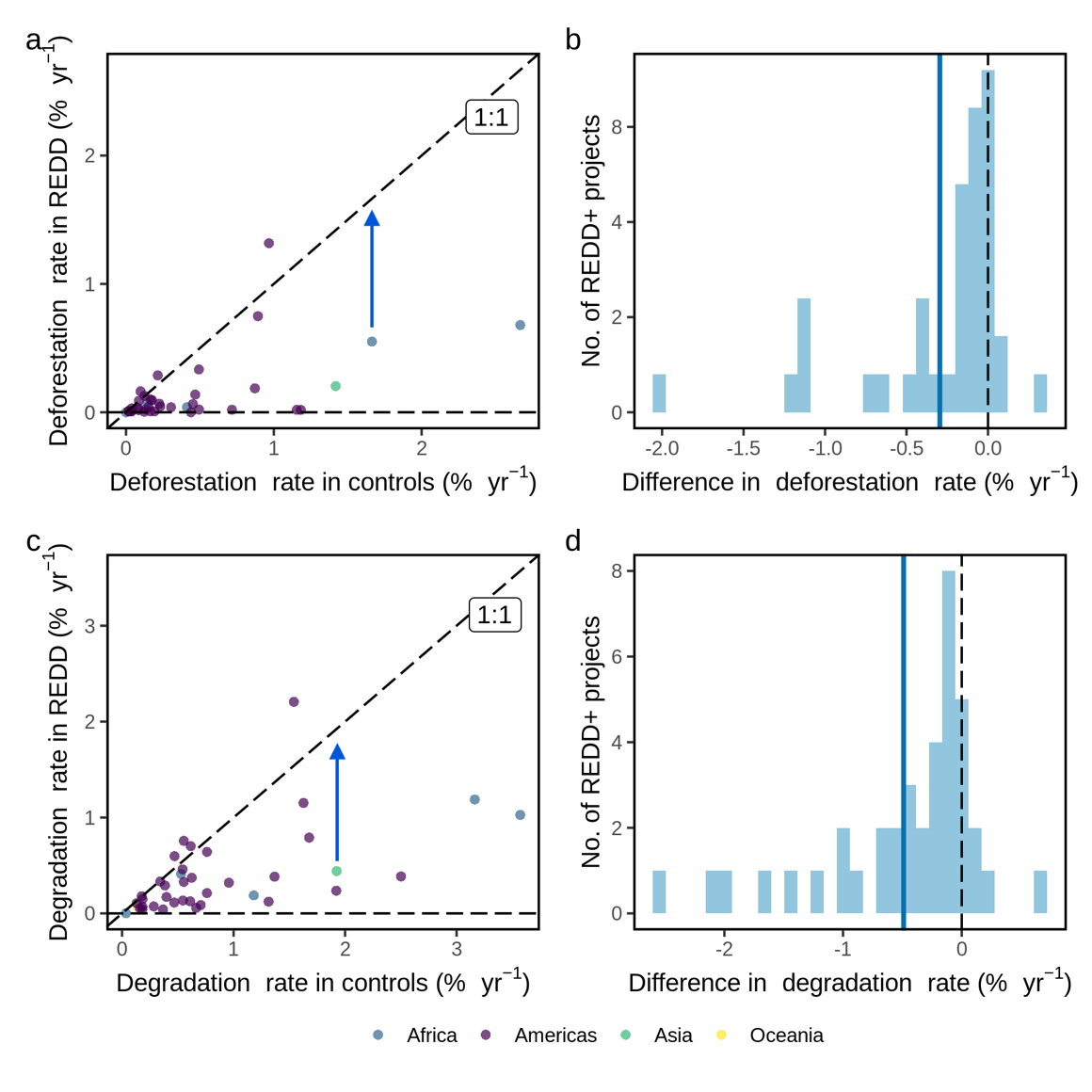


**Appendix S2.** Changes in deforestation and degradation rates resulting from REDD+ projects over their first five years of operation, excluding protected area portions. (**a**) and (**c**) scatterplots of deforestation and degradation rates in REDD+ projects versus matched control pixels; with the change in deforestation or degradation resulting from a project is given by the vertical distance between the datapoint and the diagonal 1:1 line (blue arrows); (**b**) and (**d**) histograms of the differences in deforestation and degradation rates (relative to controls), with the mean shown as a blue line and vertical dashed lines over the zero threshold.


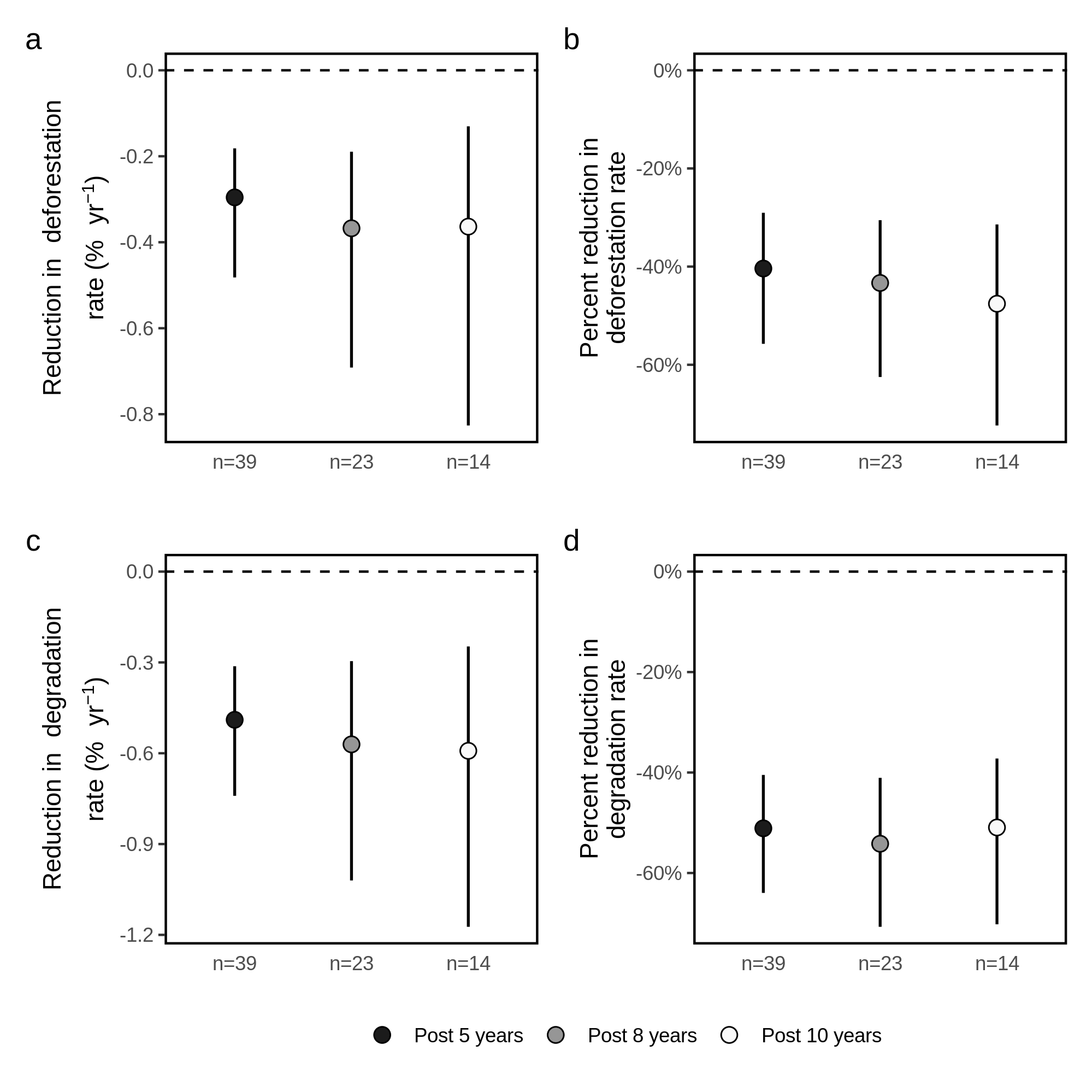


**Appendix S3.** Avoided deforestation and degradation associated with REDD+ projects considering all forested sites, excluding protected area portions, for three post implementation periods. (**a**) and (**c**) reductions in annual deforestation and degradation rates (means, with 95% confidence intervals); (**b**) and (**d**) percent reductions in deforestation and degradation rates (means, with 95% confidence intervals).


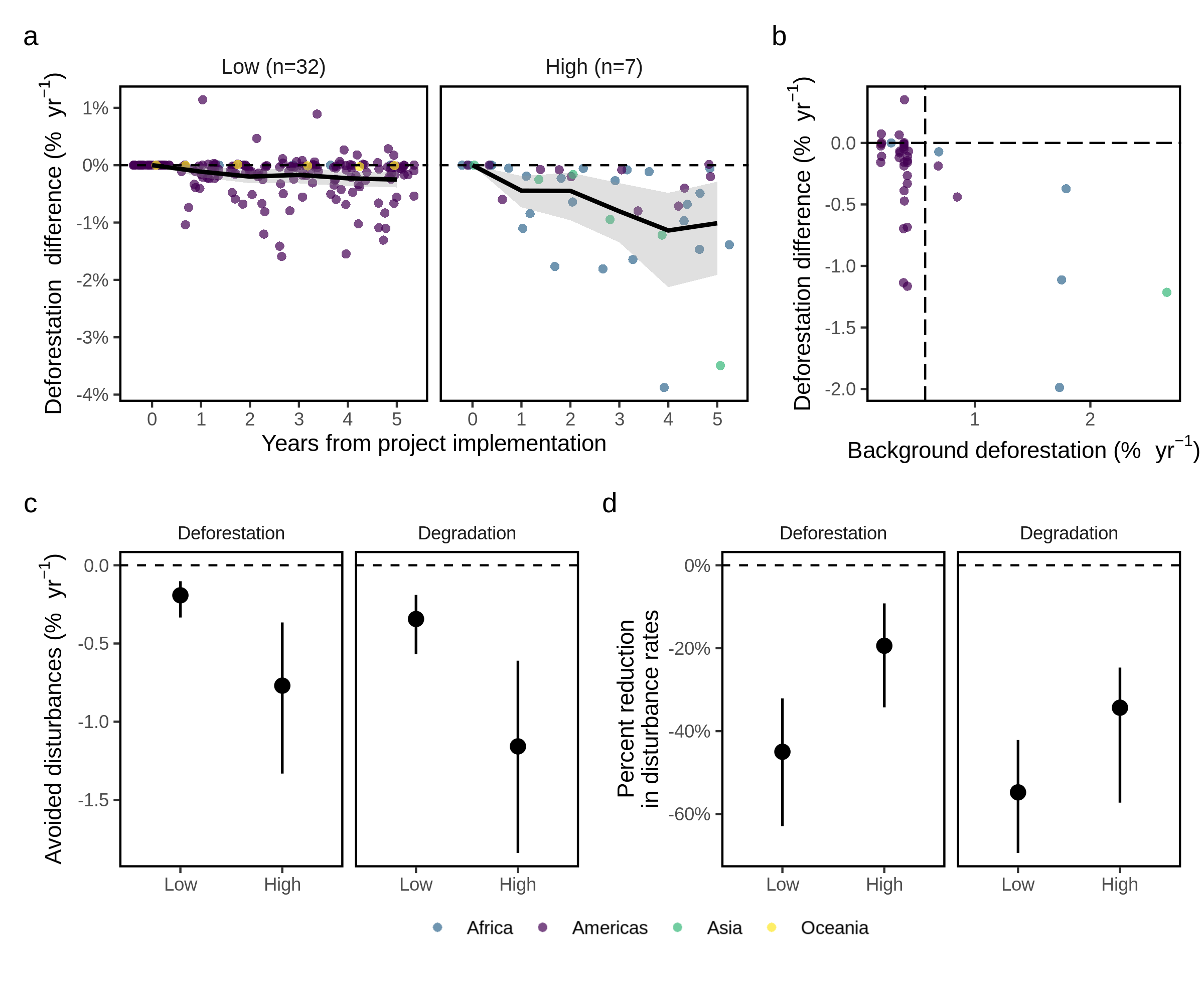


**Appendix S4.** REDD+ project effectiveness in relation to background deforestation rates, for 40 sites in humid tropical forests, excluding protected area portions. (**a**) annual differences in deforestation (with jitter; % yr^-1^) between project areas and matched controls over five years after project implementation, with a black line showing the mean annual differences and 95% CI shaded in grey; (**b**) mean differences in deforestation rates (% yr^-1^) against country-level background deforestation rates within the humid tropics (calculated for the project implementation period), with a vertical line showing the pan-tropical mean rate of deforestation (0.57% yr^-1^); (**c**) mean differences in deforestation and degradation rates within regions categorised as having low (< 0.57% yr^-1^) or high (> 0.57% yr^-1^) deforestation rates, based on the average deforestation rate across the entire humid tropics; (**d**) mean percent reductions in deforestation and degradation rates relative to controls, within regions of high and low deforestation rate. The 95% CIs displayed at **a**, **c** and **d** were estimated using non-parametric bootstrapping.

**Appendix S5.** We explored the effectiveness of REDD+ at lowering deforestation by comparing forest loss in REDD+ projects and the wider landscape in two ways. 1) Blind to overlaps with protected areas 2) excluding all areas of REDD+ or potential control areas which overlapped with protected areas. The number of REDD+ sites available for inclusion and the number of treatment and control pixels was inevitably lower for the 2nd analysis. To account for leakage, we excluded a buffer around REDD+ projects from which no pixels were sampled.

| 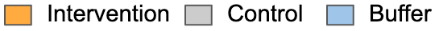 | Comparison (Treatment: T,  Control: C) | Question | Available sample  (pre-matching) |
| --- | --- | --- | --- |
| 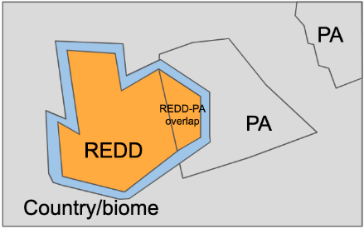  1. REDD+ vs the wider landscape | T= REDD+ sites C= Wider forested landscape (inclusive of protected areas) | 1. What is the rate of deforestation in a sample of forested pixels from REDD+ sites compared with otherwise similar pixels in the wider landscape? | 71 sites, 2.3 million T pixels, 15.8 million C pixels |
| 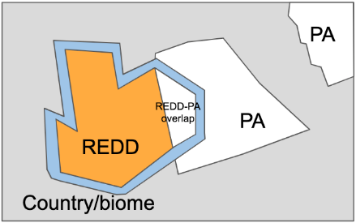  2. REDD+ vs wider non-protected landscape (i.e. excluding all PAs) | T=REDD+ sites C=Wider non-protected forested landscape (excluding protected areas) | 2. What is the rate of deforestation in a sample of forested pixels from REDD+ sites, compared with otherwise similar pixels in the wider landscape excluding protected areas. | 71 sites, 2.3 million T pixels, 12 million C pixels |


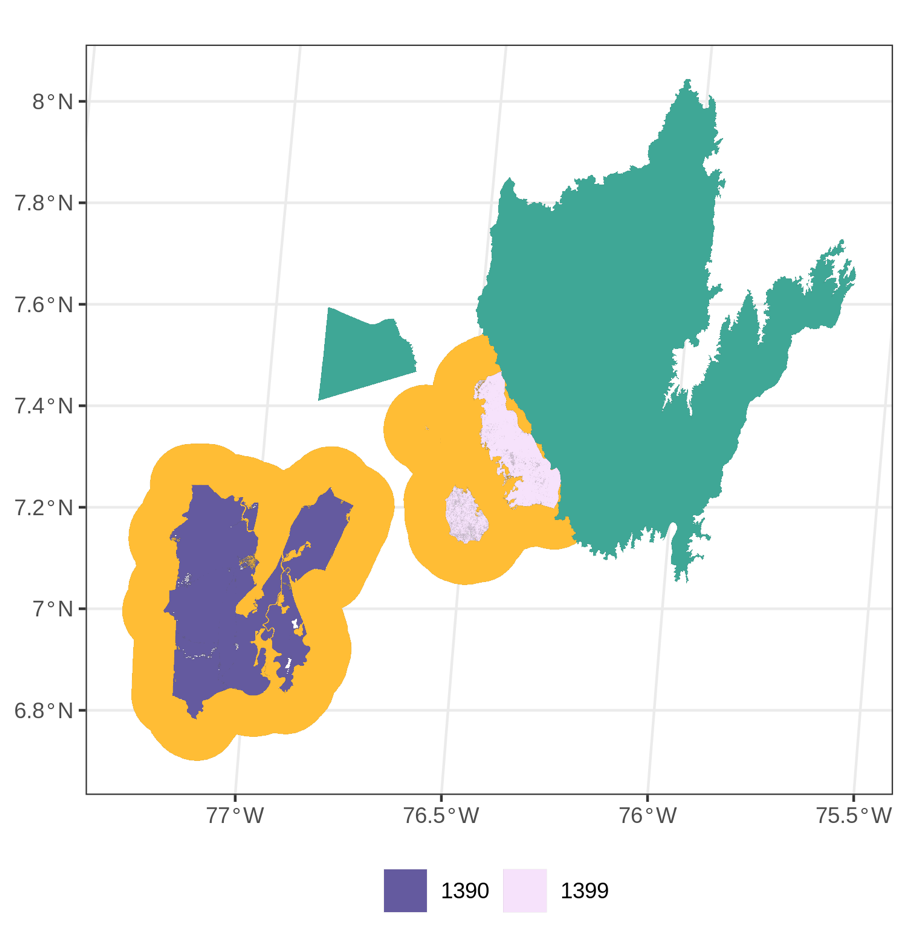


**Appendix S6:** Representation of buffer areas (in yellow) examined for leakage patterns (VCS projects 1390 and 139 shown). Overlaps with protected areas (green) were excluded from the examination.

**Appendix S7.** Covariates selected for matching. We exact matched on biome and country (shown in bold).

| Category | Rationale for inclusion | Source | Variables | Format and resolution |
| --- | --- | --- | --- | --- |
| Physical characteristics | Elevation and slope influence land use practices, with higher elevation and steeper slopes consistently associated with a lower risk of deforestation (Joppa & Pfaff 2009). | SRTM Digital Elevation Data Version 4 (Jarvis et al. 2008) | Elevation (meters) Slope (degrees) | Raster (90m2) |
| Accessibility | Accessibility to forest lands from urban centres is associated with a higher risk of deforestation (Chomitz & Thomas 2003; Rudel et al. 2009). | Global accessibility map (Weiss et al. 2018) | Time travel to population centres (seconds) | Raster (300m2) Temporal res: 2015 |
| **Bioclimatic** | The biophysical characteristics of biomes, such as temperature, precipitation and primary productivity; can influence the probability of land clearance due to agricultural suitability (Busch & Ferretti-Gallon 2017). | Terrestrial Ecoregions (Dinerstein et al. 2017). | Biome | Vector |
| **Governance /** **political economy** | Deforestation is influenced by domestic policies, governance, economic processes and markets (Umemiya et al. 2010; Ceddia et al. 2014) | Large Scale International Boundary (LSIB) dataset | Country | Vector Temporal res: 2013 |
| Proximity to deforestation | Proximity to cleared lands is consistently associated with higher deforestation risk (Busch & Ferretti-Gallon 2017). We computed the average distance to the closest deforested pixel in the 5 years prior project implementation, for each pixel included in the matching assessment. | Own analysis using the JRC Tropical Moist Forest database (Vancutsem et al. 2021) | Euclidean distance to deforested areas (meters) | Raster (30m2) |


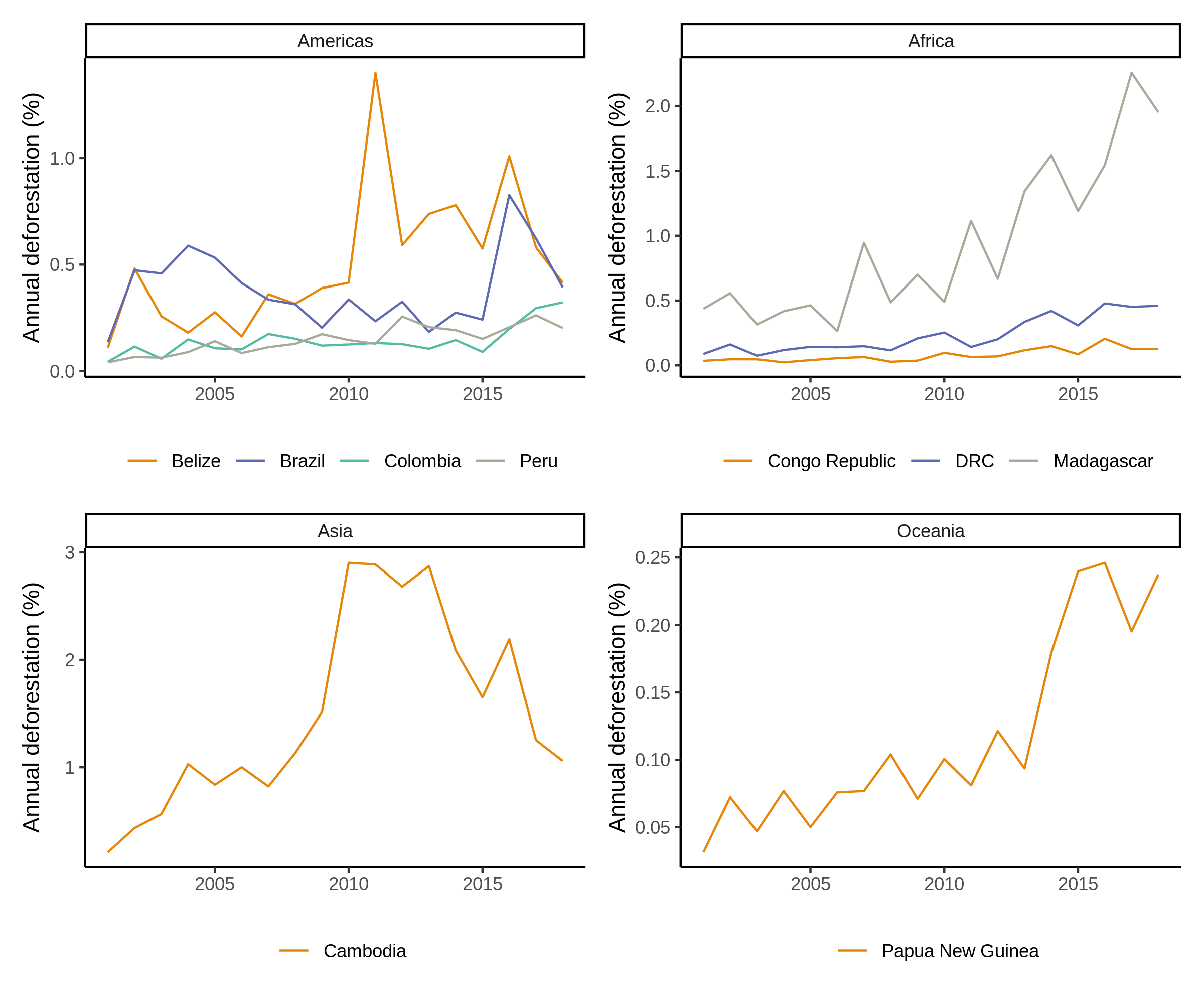


**Appendix S8:** Country-wide estimates of forest loss in the humid tropics for countries selected in the analysis. The time series were produced by subtracting deforestation and conversion to other land classes from annual estimates undisturbed and degraded Tropical Moist Forest (Vancutsem et al. 2021) across all countries spanned by our sample of 40 REDD+ projects.

**
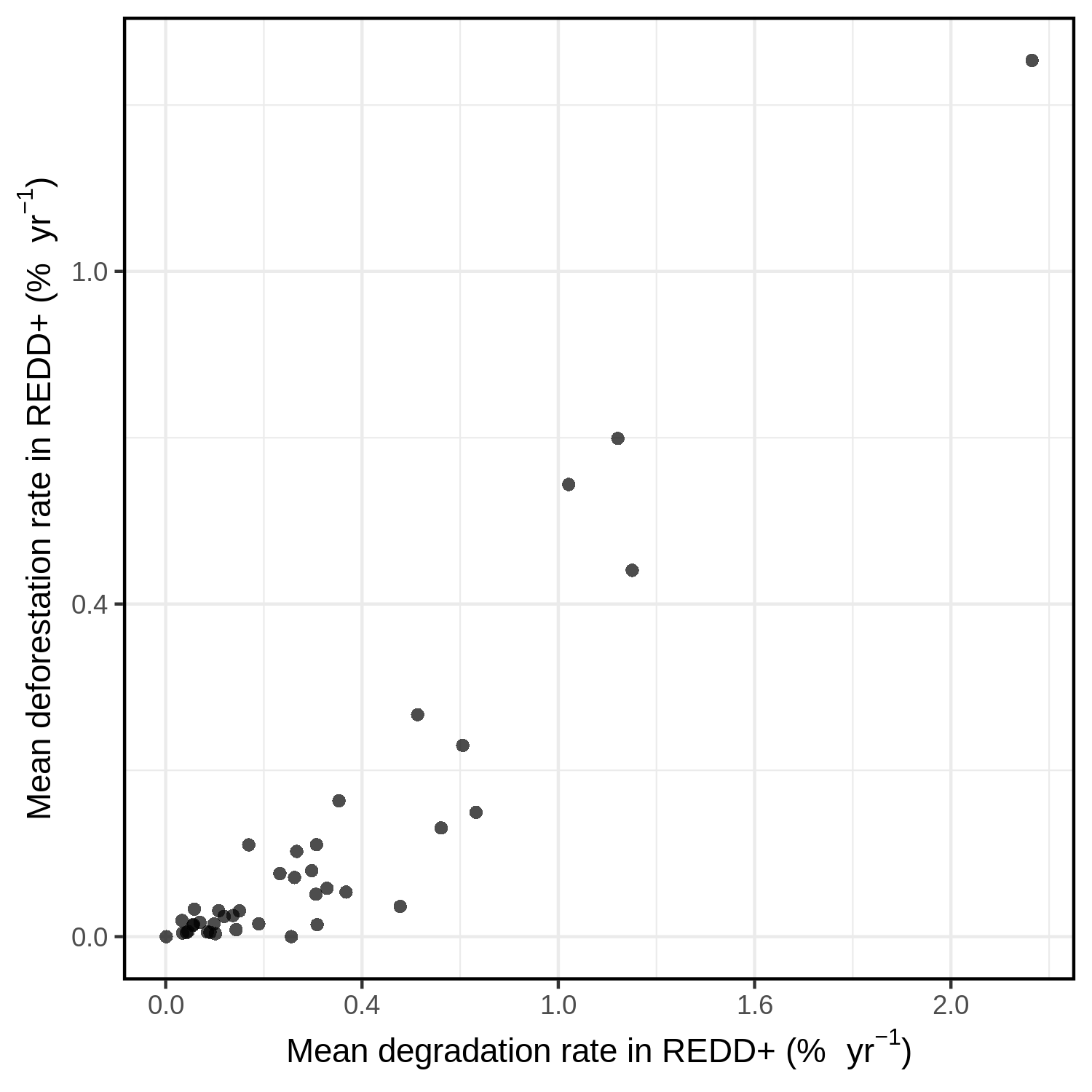
**

**Appendix S9**: Mean annual rates of forest degradation (x-axis) and deforestation (y-axis) of the examined 40 REDD+ projects, within the first 5 years of project implementation.


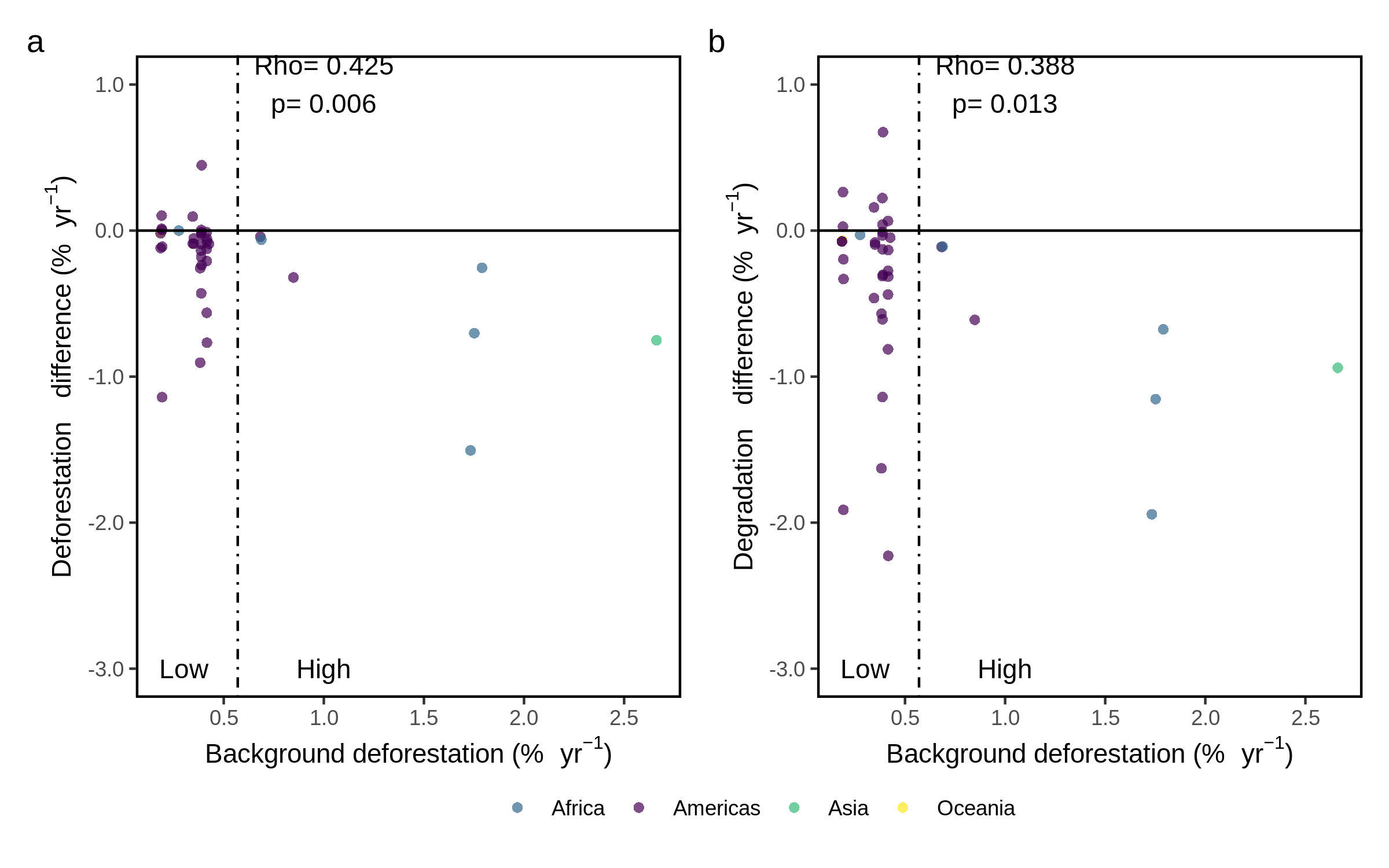
 **Appendix S10**: Mean differences in forest degradation (**a**) and deforestation (**b**) rates (% yr^-1^) against country-level background deforestation rates within the humid tropics (calculated for the project implementation period), with a vertical dotted line showing the pan-tropical mean rate of deforestation (0.57% yr^-1^)


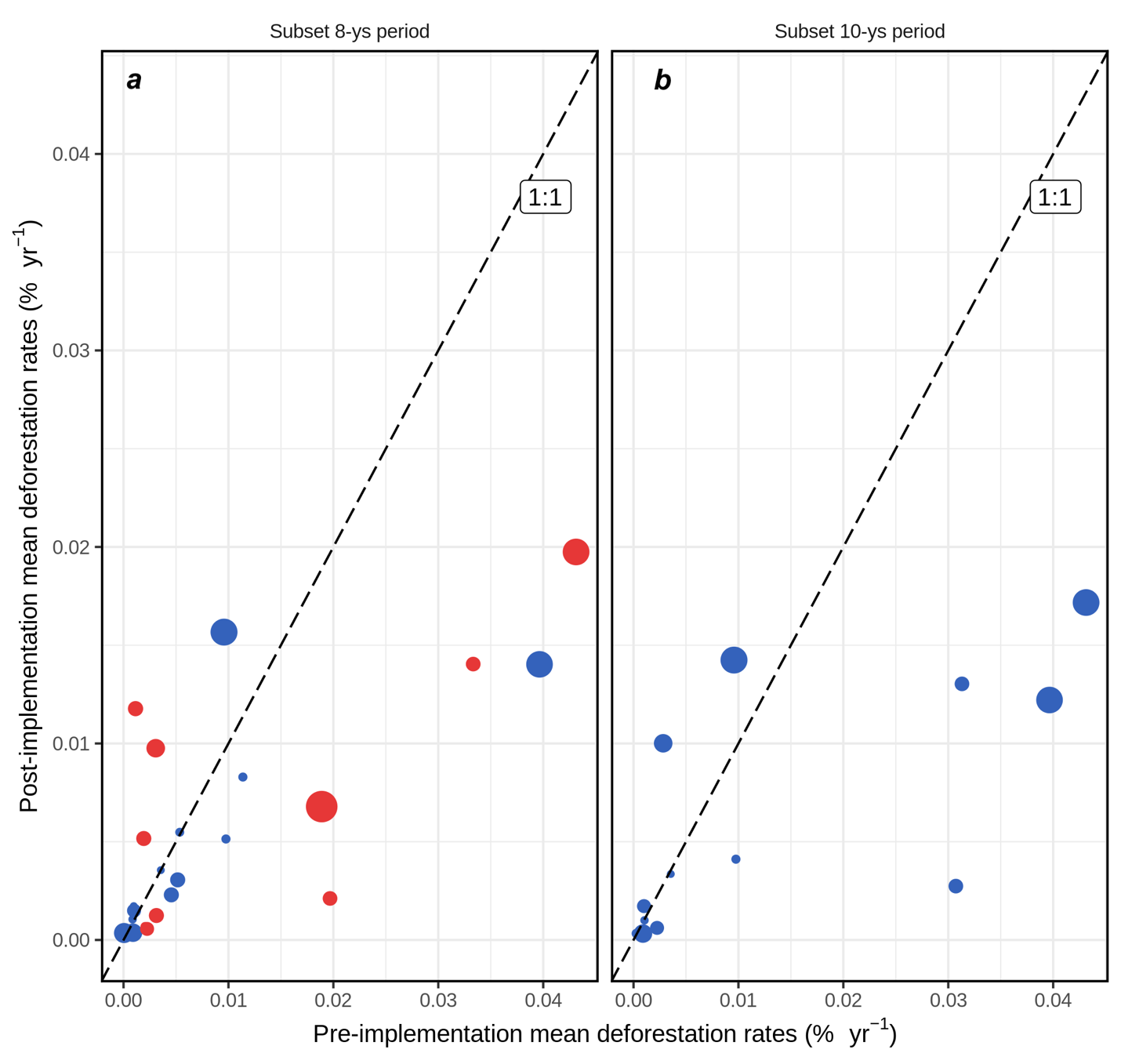
 **Appendix S11**. Evidence of deforestation leakage over eight (**a**) and ten (**b**) years after project implementation. Dots depict the mean rates of deforestation in the buffer areas before (x-axis), and after (y-axis) the commencement of projects. Red colouring indicates significant differences in annual deforestation rates before and after project implementation (bootstrapped t-tests, p < 0.05) across the 10 km boundary area. A diagonal dotted line was added to depict a theoretical 1:1 relationship between axes. Dot sizes were scaled to reflect the background deforestation rates observed at the host country within the first 5 years of project implementation


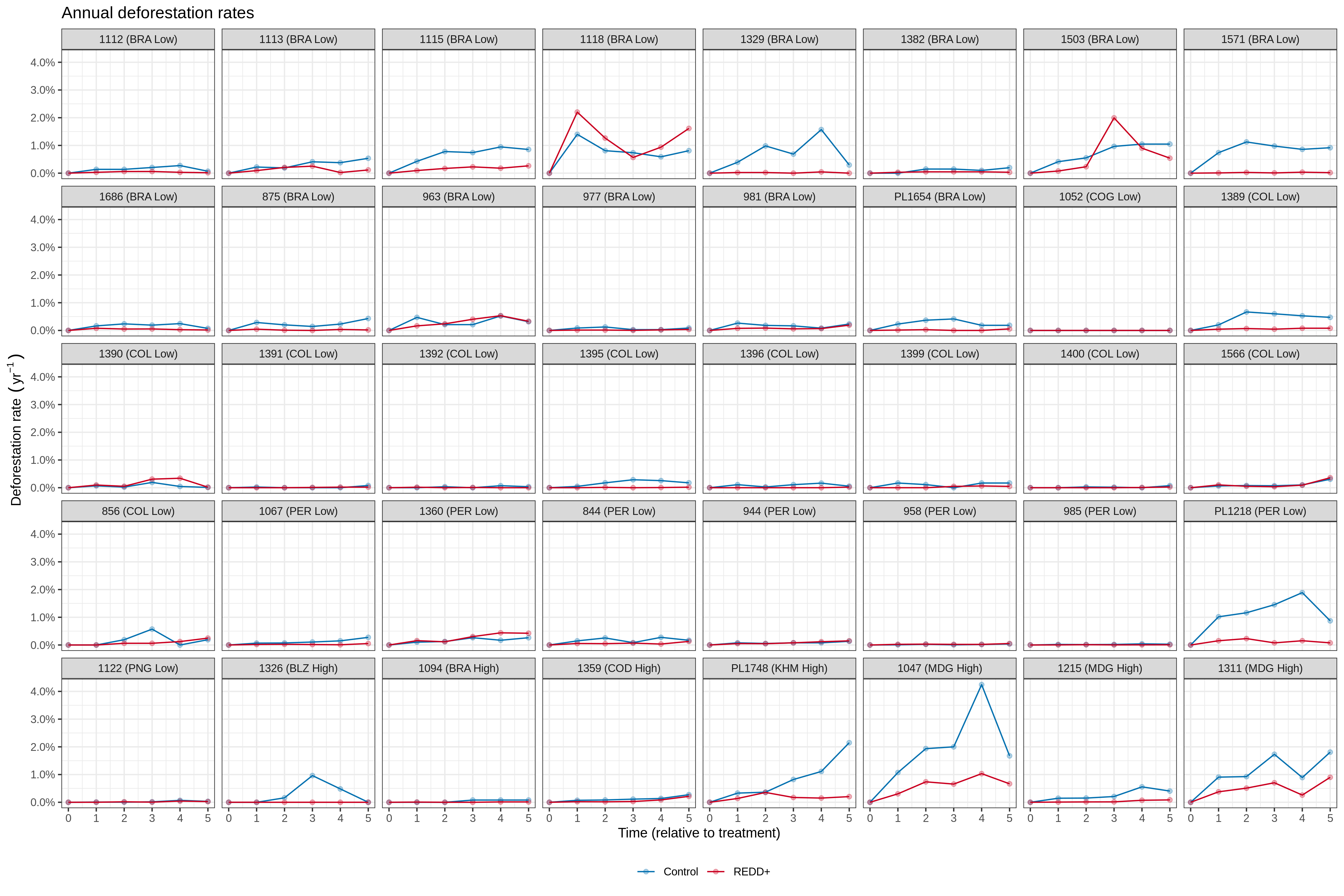


**Appendix S12**. Annual deforestation rates. Time series of the annual rates of deforestation (% yr^-1^) observed in the 40 matched project sites and their controls, five years after the implementation of REDD+. Banner indicates the project ID (see Supp. Table 3), followed by the ISO-3 country code and the classification according to the project background rates of deforestation.


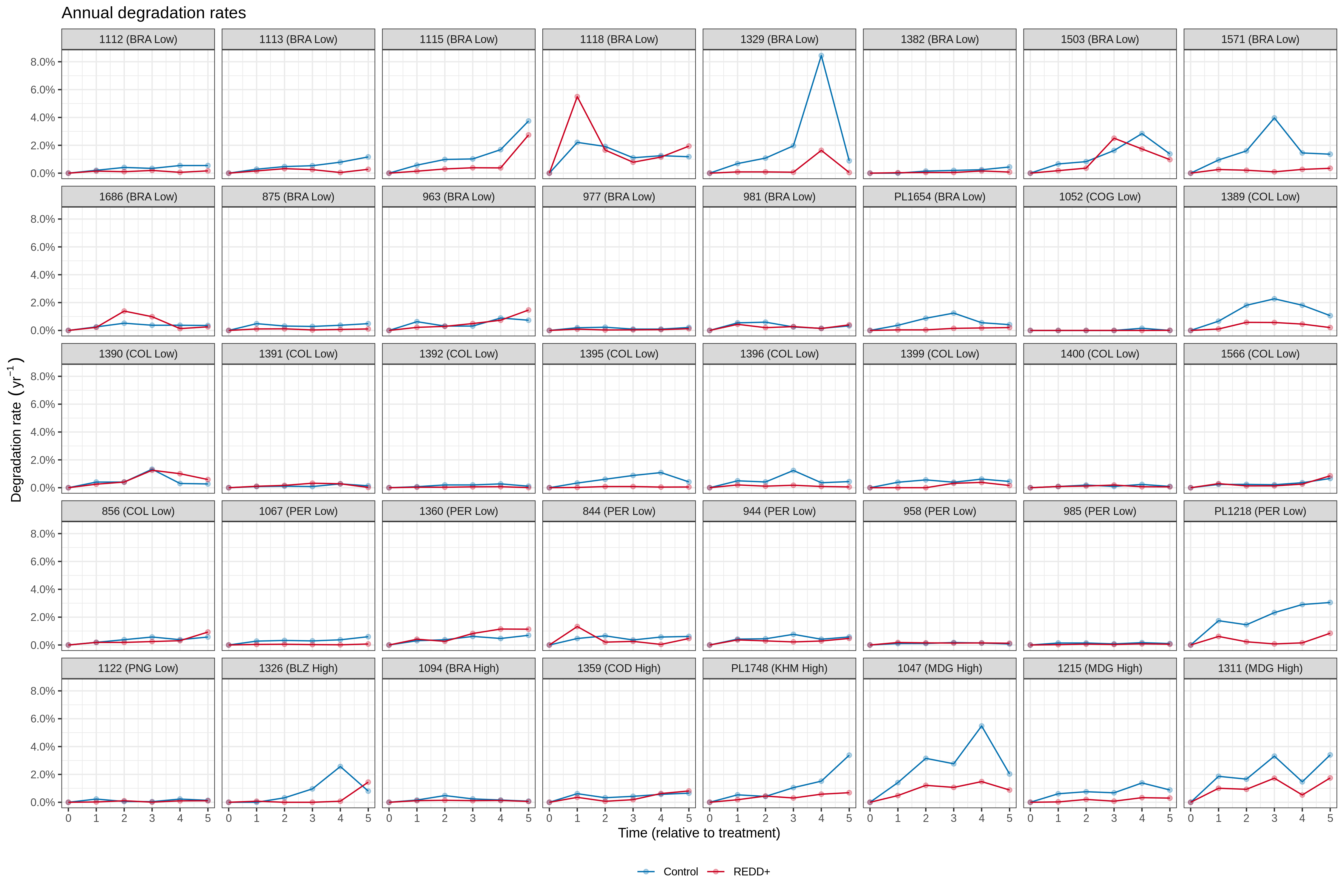


**Appendix S13**. Annual degradation rates. Time series of the annual rates of forest degradation (% yr^-1^) observed in the 40 matched project sites and their controls, five years after the implementation of REDD+. Banner indicates the project ID (see Supp. Table 4), followed by the ISO-3 country code and the classification according to the project background rates of deforestation.


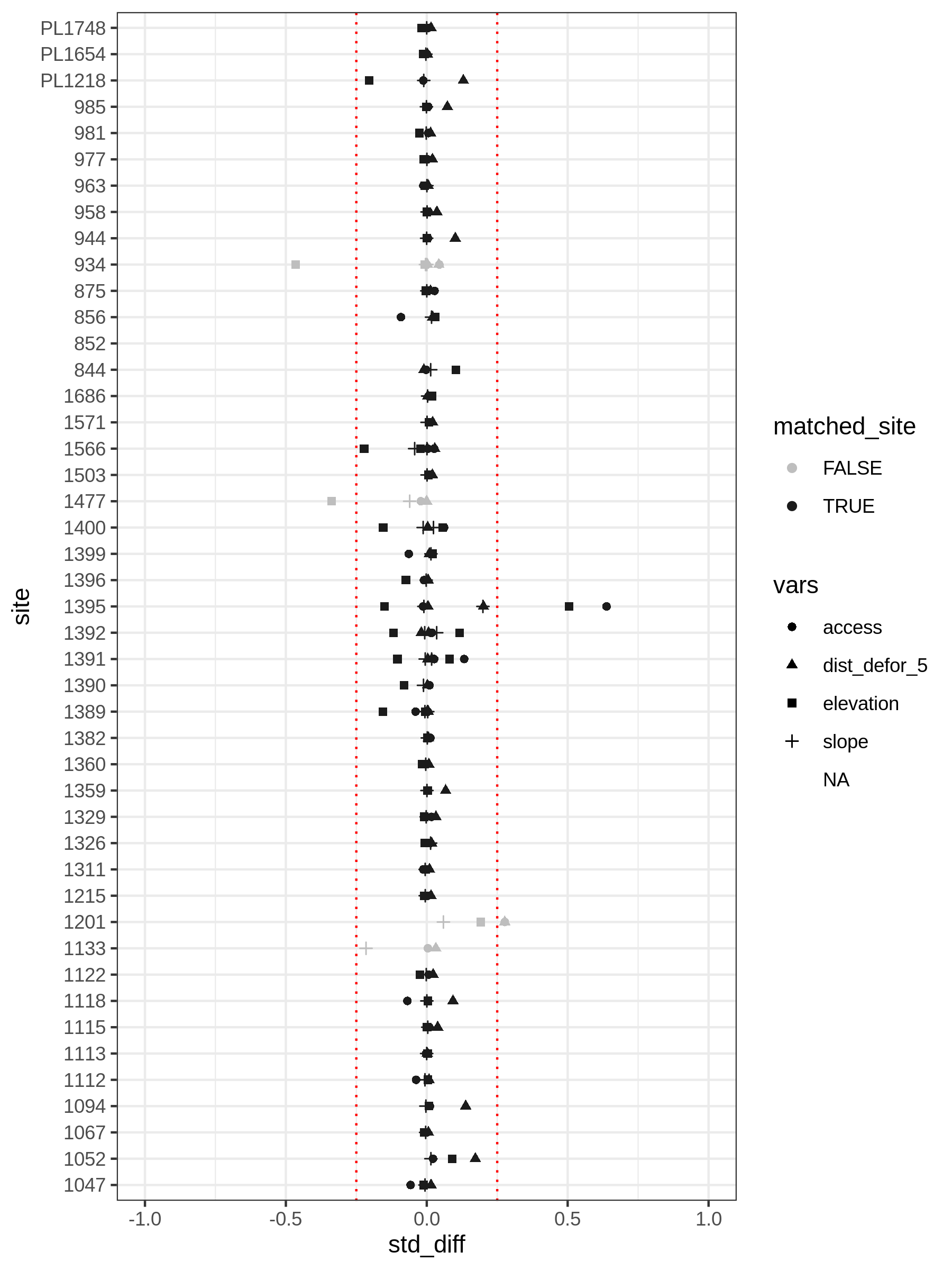


**Appendix S14**. Post-matching standardized mean differences across covariates. Sites were matched on accessibility (access), mean distance to deforestation 5 years prior to project commencement (dist_defor_5), elevation and slope; with pixels selected from the same biome and country. As 10 sites are intersected by two biomes, we constructed separate matched sets for each project/biome split (shown above as rows with 2 sets of dots). Sites were considered as successfully matched if at least 90% of the sampled pixels scored an absolute standardized mean difference of <0.25 (red lines) across all covariates. Project 1395, intersects two biomes. The pixels in one biome were not adequately matched but represent less than 10% of the total pixel count so this project is retained.


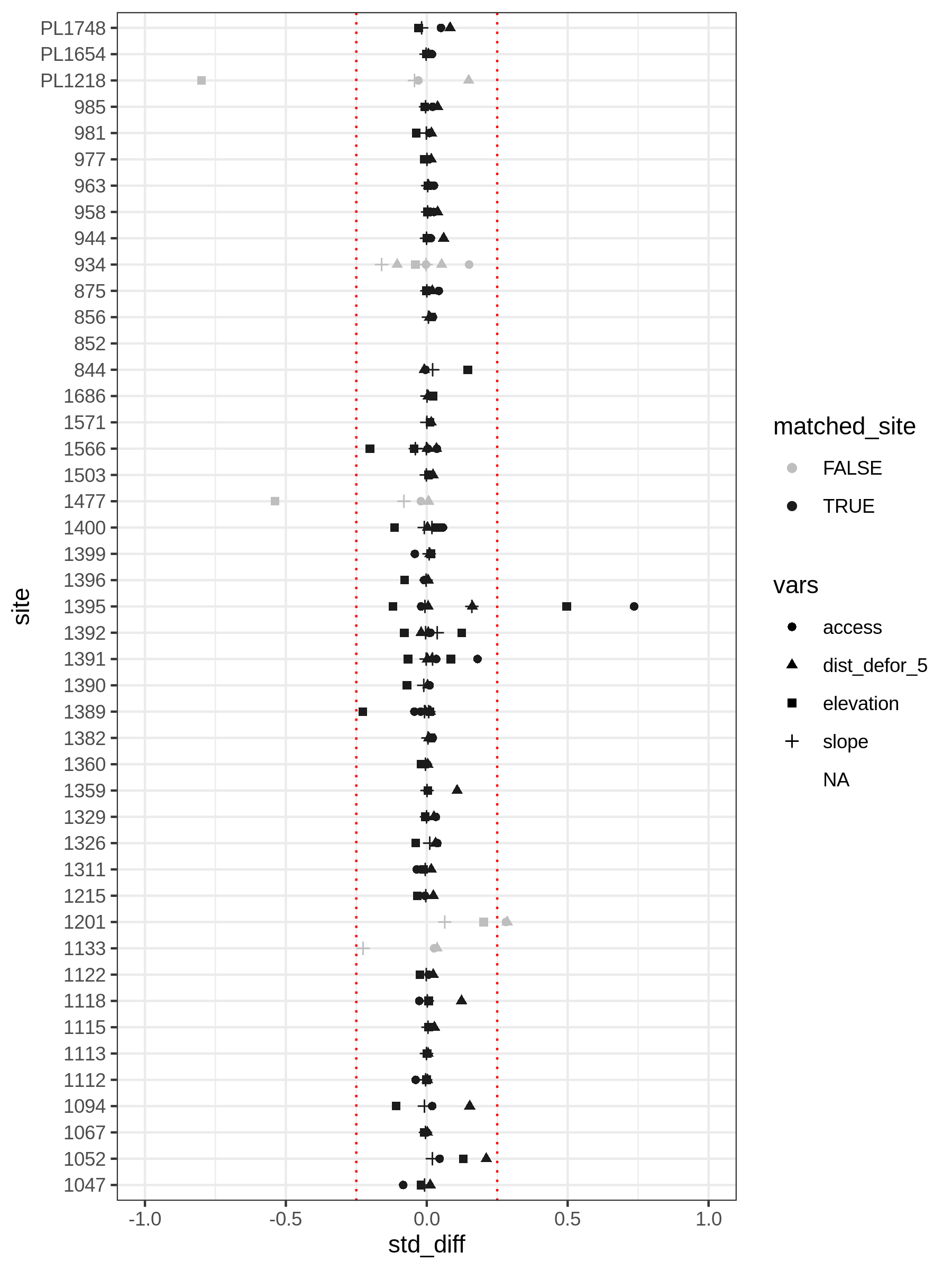


**Appendix S15**. Post-matching standardized mean differences across covariates excluding protected area portions. Sites were matched on accessibility (access), mean distance to deforestation 5 years prior to project commencement (dist_defor_5), elevation and slope; with pixels selected from the same biome and country. As 10 sites are intersected by two biomes, we constructed separate matched sets for each project/biome split (shown above as rows with 2 sets of dots). Sites were considered as successfully matched if at least 90% of the sampled pixels scored an absolute standardized mean difference of <0.25 (red lines) across all covariates. Project 1395, intersects two biomes. The pixels in one biome were not adequately matched but represent less than 10% of the total pixel count so this project is retained.


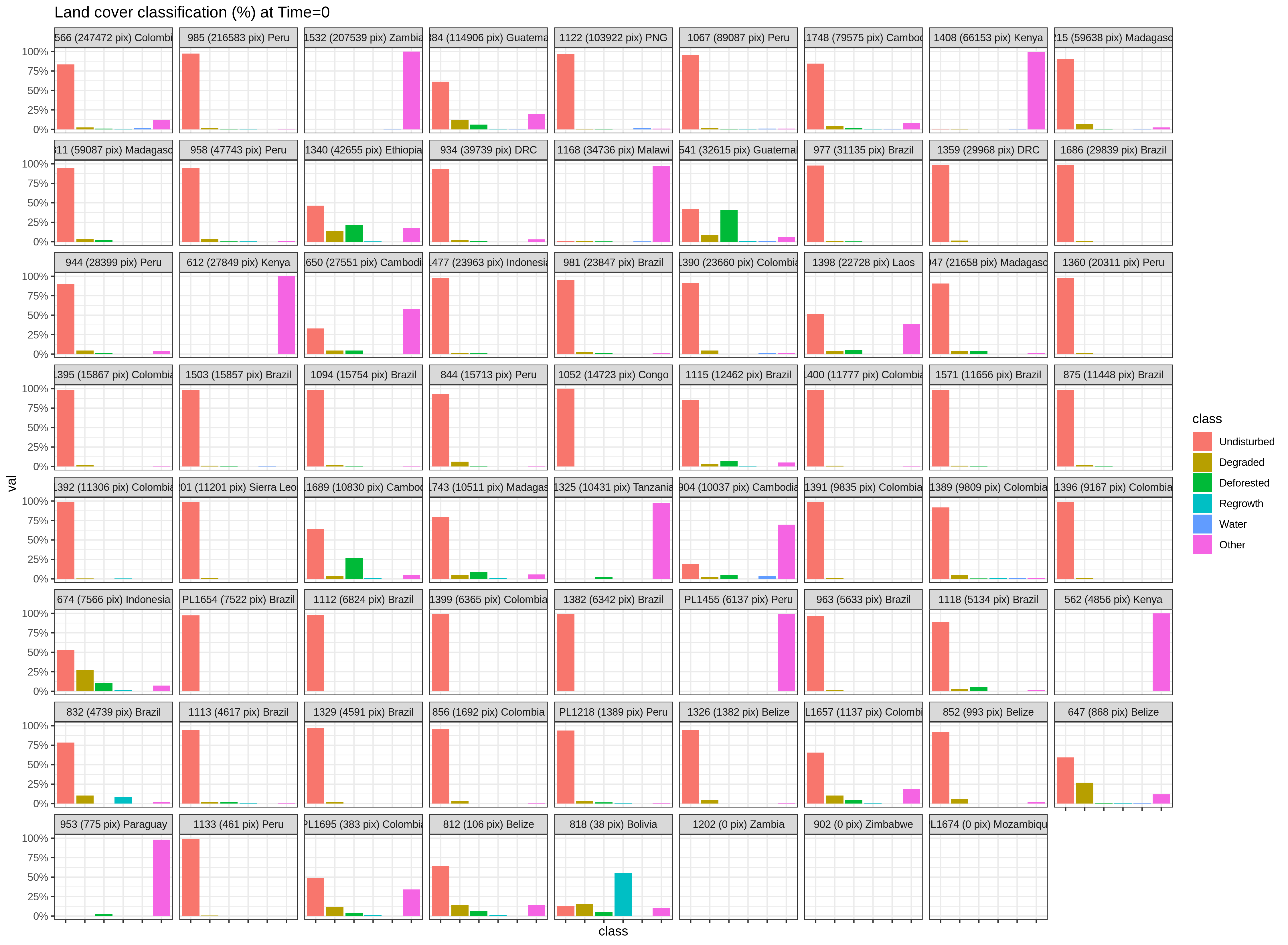


**Appendix S16**. Distribution of land cover classes at the project starting date**.** The banner indicates the project ID, followed by the total count of pixels within the boundaries and the host country, for the 71 sites for which we were able to collect REDD+ boundary information. Projects with at least 80% of evergreen forest cover at the starting date were included in the analyses.

**Appendix S17**. Logistic regression (binary response variable, 0 = VCS REDD+ sites excluded from the analysis n=31; 1 = sites included in the analysis n=40) to assess the probability of a project being selected due to covariate characteristics. Statistically significant effects (P<0.05) indicate the probability of a selection bias in our sample (shown in bold). The p-value were adjusted through a post-hoc Bonferroni correction.

|  | Estimate | Std. Error | z value | P value |
| --- | --- | --- | --- | --- |
| (Intercept) | -7.79 | 2.83 | -2.76 | 0.04 |
| **Time travel to population centres (seconds) in 2015** | **0.00** | **0.00** | **2.99** | **0.02** |
| Elevation (m) | 0.00 | 0.00 | -0.47 | 1 |
| Slope (^o^) | 0.00 | 0.08 | 0.03 | 1 |
| Human Development Index (2010-2018) | 9.22 | 3.71 | 2.49 | 0.06 |
| Project area (km) | 0.00 | 0.00 | 0.14 | 1 |
| Distance to recent forest clearing by starting date (m) | 0.00 | 0.00 | -0.08 | 1 |

**Appendix S18**. Characteristics of examined projects

| ID | Name | Country and ISO 3 code | Start year | Area (ha) | PA overlap (%) | Mean deforestation REDD+ (y^-1^) | Mean deforestation control (y^-1^) | Mean degradation REDD+ (y^-1^) | Mean degradation control (y^-1^) | Deforestation at host country | Threat group | Undisturbed forest cover (%) at starting date |
| --- | --- | --- | --- | --- | --- | --- | --- | --- | --- | --- | --- | --- |
| 1326 | Laguna Seca Forest Carbon Project | Belize (BLZ) | 2011 | 8723 | 0.2 | 0 | 0.32 | 0.32 | 0.93 | 0.8 | High | 94.9 |
| 1094 | Ecomapua Amazon REDD Project | Brazil (BRA) | 2002 | 99149 | 99.8 | 0.01 | 0.05 | 0.11 | 0.22 | 0.7 | High | 97.8 |
| 981 | ADPML Portel-Para REDD Project | Brazil (BRA) | 2008 | 150204 | 0 | 0.09 | 0.18 | 0.29 | 0.37 | 0.4 | Low | 94.6 |
| 977 | RMDLT Portel-Para REDD Project | Brazil (BRA) | 2008 | 211131 | 0 | 0.02 | 0.07 | 0.07 | 0.16 | 0.4 | Low | 98 |
| 1118 | Suruí Forest Carbon Project | Brazil (BRA) | 2009 | 33626 | 100 | 1.32 | 0.87 | 2.21 | 1.53 | 0.4 | Low | 89.3 |
| 875 | Florestal Santa Maria Project | Brazil (BRA) | 2009 | 72120 | 0 | 0.02 | 0.26 | 0.09 | 0.39 | 0.4 | Low | 98 |
| 1113 | The Valparaiso Project | Brazil (BRA) | 2011 | 29195 | 0 | 0.14 | 0.35 | 0.21 | 0.65 | 0.4 | Low | 94.2 |
| 963 | The Purus Project | Brazil (BRA) | 2011 | 35746 | 7.1 | 0.33 | 0.35 | 0.64 | 0.58 | 0.4 | Low | 96.8 |
| 1112 | The Russas Project | Brazil (BRA) | 2011 | 42788 | 0 | 0.04 | 0.16 | 0.13 | 0.41 | 0.4 | Low | 97.8 |
| 1115 | Jari/Amapá REDD+ Project | Brazil (BRA) | 2011 | 78232 | 1.4 | 0.19 | 0.75 | 0.79 | 1.6 | 0.4 | Low | 85.1 |
| 1329 | Maísa REDD+ Project | Brazil (BRA) | 2012 | 30242 | 0 | 0.02 | 0.79 | 0.39 | 2.61 | 0.4 | Low | 97.1 |
| 1382 | The Envira Amazonia Project - A Tropical Forest Conservation Project in Acre | Brazil (BRA) | 2012 | 39749 | 0 | 0.04 | 0.12 | 0.07 | 0.21 | 0.4 | Low | 99.2 |
| 1503 | Resex Rio Preto-Jacundá REDD+ Project | Brazil (BRA) | 2012 | 101592 | 99.1 | 0.75 | 0.81 | 1.15 | 1.47 | 0.4 | Low | 98 |
| PL1654 | Fortaleza Ituxi REDD Project | Brazil (BRA) | 2013 | 47327 | 0 | 0.02 | 0.28 | 0.12 | 0.69 | 0.4 | Low | 97.3 |
| 1571 | Manoa REDD+ Project | Brazil (BRA) | 2013 | 73620 | 0.1 | 0.02 | 0.92 | 0.24 | 1.87 | 0.4 | Low | 98.5 |
| 1686 | Agrocortex REDD Project | Brazil (BRA) | 2014 | 187740 | 0 | 0.05 | 0.18 | 0.6 | 0.38 | 0.4 | Low | 98.9 |
| PL1748 | Southern Cardamoms REDD+ Project | Cambodia (KHM) | 2010 | 500452 | 29.6 | 0.2 | 0.96 | 0.44 | 1.38 | 2.7 | High | 84.3 |
| 856 | The Chocó-Darién Conservation Corridor REDD Project | Colombia (COL) | 2010 | 10687 | 95.7 | 0.1 | 0.19 | 0.37 | 0.42 | 0.4 | Low | 95.4 |
| 1399 | Mutatá REDD+ Project | Colombia (COL) | 2013 | 40471 | 0.7 | 0.03 | 0.12 | 0.17 | 0.48 | 0.4 | Low | 99.1 |
| 1391 | SUPP REDD+ Project | Colombia (COL) | 2013 | 54210 | 49.7 | 0.01 | 0.02 | 0.18 | 0.14 | 0.4 | Low | 98.5 |
| 1389 | Acapa -­ Bajo Mira Y Frontera REDD+ Project | Colombia (COL) | 2013 | 55448 | 11.5 | 0.06 | 0.49 | 0.38 | 1.52 | 0.4 | Low | 91.8 |
| 1400 | Concosta REDD+ Project | Colombia (COL) | 2013 | 64997 | 49.7 | 0.01 | 0.02 | 0.11 | 0.14 | 0.4 | Low | 98.2 |
| 1392 | Cajambre REDD+ Project | Colombia (COL) | 2013 | 65845 | 1.4 | 0.01 | 0.03 | 0.04 | 0.17 | 0.4 | Low | 98.5 |
| 1395 | Bajo Calima y Bahía Málaga (BCBM) REDD+ Project | Colombia (COL) | 2013 | 91971 | 27.3 | 0.01 | 0.19 | 0.06 | 0.67 | 0.4 | Low | 97.7 |
| 1566 | REDD+ Project Resguardo Indigena Unificado Selva de Mataven | Colombia (COL) | 2013 | 1557190 | 0 | 0.13 | 0.12 | 0.33 | 0.34 | 0.4 | Low | 83.5 |
| 1396 | Rio Pepe y ACABA REDD+ Project | Colombia (COL) | 2014 | 61132 | 0 | 0 | 0.09 | 0.13 | 0.59 | 0.3 | Low | 98.3 |
| 1390 | Carmen del Darién REDD+ Project | Colombia (COL) | 2014 | 134243 | 0 | 0.16 | 0.07 | 0.7 | 0.54 | 0.3 | Low | 91.6 |
| 1052 | North Pikounda REDD+ | Congo (COG) | 2012 | 92735 | 2.8 | 0 | 0 | 0 | 0.03 | 0.3 | Low | 100 |
| 1359 | Isangi REDD+ Project | Congo DRC (COD) | 2009 | 199421 | 0 | 0.07 | 0.14 | 0.41 | 0.52 | 0.7 | High | 98.1 |
| 1215 | The Makira Forest Protected Area in Madagascar | Madagascar (MDG) | 2005 | 374708 | 0 | 0.04 | 0.29 | 0.19 | 0.86 | 1.8 | High | 90 |
| 1047 | Carbon Emissions Reduction Project in the Forest Corridor Ambositra-Vondrozo (COFAV) | Madagascar (MDG) | 2007 | 144336 | 0.6 | 0.68 | 2.19 | 1.03 | 2.97 | 1.7 | High | 90.8 |
| 1311 | Carbon Emissions Reduction Project in the Corridor Ankeniheny-Zahamena (CAZ) Protected Area | Madagascar (MDG) | 2008 | 392643 | 0.8 | 0.55 | 1.25 | 1.19 | 2.34 | 1.8 | High | 94.5 |
| 1122 | April Salumei REDD Project | Papua New Guinea (PNG) | 2009 | 653933 | 35 | 0.02 | 0.03 | 0.07 | 0.14 | 0.2 | Low | 96.6 |
| 844 | Madre de Dios Amazon REDD Project | Peru (PER) | 2006 | 98539 | 0 | 0.07 | 0.19 | 0.46 | 0.53 | 0.2 | Low | 93.1 |
| 985 | Cordillera Azul National Park REDD project | Peru (PER) | 2006 | 1362722 | 100 | 0.01 | 0.02 | 0.05 | 0.13 | 0.2 | Low | 97.5 |
| 944 | Alto Mayo Conservation Initiative | Peru (PER) | 2007 | 178704 | 98 | 0.09 | 0.08 | 0.33 | 0.52 | 0.2 | Low | 89.5 |
| 1067 | Reduction of deforestation and degradation in Tambopata National Reserve and Bahuaja-Sonene National Park within the area of Madre de Dios region | Peru (PER) | 2008 | 565849 | 99.6 | 0.02 | 0.13 | 0.04 | 0.37 | 0.2 | Low | 95.7 |
| 1360 | Forest Management to reduce deforestation and degradation in Shipibo Conibo and Cacataibo Indigenous communities of Ucayali region | Peru (PER) | 2010 | 129260 | 0 | 0.29 | 0.18 | 0.76 | 0.49 | 0.2 | Low | 97.6 |
| 958 | Biocorredor Martín Sagrado REDD+ project | Peru (PER) | 2010 | 300275 | 0 | 0.03 | 0.02 | 0.15 | 0.12 | 0.2 | Low | 95 |
| PL1218 | Evio Kuiñaji Ese´Eja Cuana, To Mitigate Climate Change, Madre de Dios | Peru (PER) | 2011 | 8724 | 18.4 | 0.14 | 1.28 | 0.38 | 2.3 | 0.2 | Low | 93.7 |

### References

Busch J, Ferretti-Gallon K. 2017. What Drives Deforestation and What Stops It? A Meta-Analysis. Review of Environmental Economics and Policy **11**:3–23.

Ceddia MG, Bardsley NO, Gomez-y-Paloma S, Sedlacek S. 2014. Governance, agricultural intensification, and land sparing in tropical South America. Proceedings of the National Academy of Sciences **111**:7242–7247.

Chomitz KM, Thomas TS. 2003. Determinants of Land Use in Amazônia: A Fine-Scale Spatial Analysis. American Journal of Agricultural Economics **85**:1016–1028.

Dinerstein E et al. 2017. An Ecoregion-Based Approach to Protecting Half the Terrestrial Realm. Bioscience **67**:534–545.

Jarvis A, Reuter HI, Nelson A, Guevara E. 2008. Hole-filled SRTM for the Globe. v4. CGIAR. Available from http://srtm.csi.cgiar.org.

Joppa LN, Pfaff A. 2009. High and far: biases in the location of protected areas. PLoS One **4**:e8273.

Rudel TK et al. 2009. Agricultural intensification and changes in cultivated areas, 1970-2005. Proc. Natl. Acad. Sci. U. S. A. **106**:20675–20680.

Umemiya C, Rametsteiner E, Kraxner F. 2010. Quantifying the impacts of the quality of governance on deforestation. Environmental Science & Policy **13**:695–701.

Vancutsem C, Achard F, Pekel J-F, Vieilledent G, Carboni S, Simonetti D, Gallego J, Aragão LEOC, Nasi R. 2021. Long-term (1990–2019) monitoring of forest cover changes in the humid tropics. Science Advances **7**:eabe1603.

Weiss DJ et al. 2018. A global map of travel time to cities to assess inequalities in accessibility in 2015. Nature **553**:333–336.
